# Single-Cell RNA Sequencing of Coronary Perivascular Adipose Tissue From End-Stage Heart Failure Patients Identifies *SPP1*^+^ Macrophage Subpopulation as a Target for Alleviating Fibrosis

**DOI:** 10.1161/ATVBAHA.123.319828

**Published:** 2023-09-14

**Authors:** Mengxia Fu, Songren Shu, Zhiming Peng, Xiaorui Liu, Xiao Chen, Zhiwei Zeng, Yicheng Yang, Hao Cui, Ruojin Zhao, Xiaohu Wang, Leilei Du, Min Wu, Wei Feng, Jiangping Song

**Affiliations:** State Key Laboratory of Cardiovascular Disease, Fuwai Hospital, National Center for Cardiovascular Diseases (M.F., S.S., X.L., X.C., Z.Z., Y.Y., H.C., R.Z., X.W., W.F., J.S.), Chinese Academy of Medical Sciences and Peking Union Medical College, Beijing, China.; Department of Orthopedics, Peking Union Medical College Hospital (Z.P.), Chinese Academy of Medical Sciences and Peking Union Medical College, Beijing, China.; Department of Cardiovascular Surgery, Fuwai Hospital, National Center for Cardiovascular Diseases (W.F., J.S.), Chinese Academy of Medical Sciences and Peking Union Medical College, Beijing, China.; Galactophore Department, Galactophore Center, Beijing Shijitan Hospital (M.F., M.W.), Capital Medical University, China.; Laboratory of Cardiovascular Science, Beijing Clinical Research Institute, Beijing Friendship Hospital (L.D.), Capital Medical University, China.; The Cardiomyopathy Research Group at Fuwai Hospital, China (S.S., X.L., X.C., H.C., R.Z., X.W., J.S.).; Shenzhen Key Laboratory of Cardiovascular Disease, Fuwai Hospital Chinese Academy of Medical Sciences, China (J.S.).

**Keywords:** atherosclerosis, fibrosis, macrophages, single-cell gene expression analysis, stromal vascular fraction

## Abstract

**BACKGROUND:**

Perivascular adipose tissue (PVAT) is vital for vascular homeostasis, and PVAT dysfunction is associated with increased atherosclerotic plaque burden. But the mechanisms underlining coronary PVAT dysfunction in coronary atherosclerosis remain elusive.

**METHODS:**

We performed single-cell RNA sequencing of the stromal vascular fraction of coronary PVAT from 3 groups of heart transplant recipients with end-stage heart failure, including 3 patients with nonobstructive coronary atherosclerosis, 3 patients with obstructive coronary artery atherosclerosis, and 4 nonatherosclerosis control subjects. Bioinformatics was used to annotate the cellular populations, depict the cellular developmental trajectories and interactions, and explore the differences among 3 groups of coronary PVAT at the cellular and molecular levels. Pathological staining, quantitative real-time polymerase chain reaction, and in vitro studies were performed to validate the key findings.

**RESULTS:**

Ten cell types were identified among 67 936 cells from human coronary PVAT. Several cellular subpopulations, including *SPP1*^+^ (secreted phosphoprotein 1) macrophages and profibrotic fibroadipogenic progenitor cells, were accumulated in PVAT surrounding atherosclerotic coronary arteries compared with nonatherosclerosis coronary arteries. The fibrosis percentage was increased in PVAT surrounding atherosclerotic coronary arteries, and it was positively associated with the grade of coronary artery stenosis. Cellular interaction analysis suggested OPN (osteopontin) secreted by *SPP1*^+^ macrophages interacted with CD44 (cluster of differentiation 44)/integrin on fibroadipogenic progenitor cells. Strikingly, correlation analyses uncovered that higher level of SPP1 in PVAT correlates with a more severe fibrosis degree and a higher coronary stenosis grade. In vitro studies showed that conditioned medium from atherosclerotic coronary PVAT promoted the migration and proliferation of fibroadipogenic progenitor cells, while such effect was prevented by blocking CD44 or integrin.

**CONCLUSIONS:**

*SPP1*^+^ macrophages accumulated in the PVAT surrounding atherosclerotic coronary arteries, and they promoted the migration and proliferation of fibroadipogenic progenitor cells via OPN-CD44/integrin interaction and thus aggravated the fibrosis of coronary PVAT, which was positively correlated to the coronary stenosis burden. Therefore, *SPP1*^+^ macrophages in coronary PVAT may participate in the progression of coronary atherosclerosis.

**Highlights**

- Single-cell RNA sequencing reveals the coronary perivascular adipose tissue cell atlas and suggests novel functional interactions among resident cell subpopulations.
- This study provides insight into the dynamic roles of cellular subpopulations from coronary perivascular adipose tissue in the progression of atherosclerosis.
- Our results identified potential roles of *SPP1*^+^ (secreted phosphoprotein 1) macrophage of perivascular adipose tissue in coronary atherosclerosis.

Coronary heart disease (CHD) remains the predominant type of cardiovascular disease worldwide, causing a substantial medical and economic burden.^1^ The characteristic pathological features of CHD include the formation and rupture of atherosclerotic plaques in the early and advanced stages, respectively.^2,3^ Therefore, preventing atherosclerotic plaque progression is one of the main strategies to avoid adverse cardiovascular events.^4^ However, evidence to support the effectiveness of lipid-lowering drugs to prevent plaque progression is limited at present.^5^ One key reason for this dilemma is the incomplete understanding of the pathogenesis of coronary atherosclerosis.

Coronary perivascular adipose tissue (PVAT) dysfunction is closely related to the progression of coronary atherosclerosis. Several cross-sectional studies have indicated that coronary PVAT attenuation could distinguish different stages of CHD.^6,7^ Moreover, a prospective cohort study showed that the radiomics signature of coronary PVAT at baseline could predict 3-year acute coronary syndrome in patients with suspected CHD.^8^ Basic research suggests that deletion of PPARγ (peroxisome proliferator-activated receptor gamma) in PVAT enhances atherosclerosis in mice,^9^ while transplantation of PVAT on the carotid artery increased vascular remodeling after a wire-induced injury in LDL^−/−^ mice.^10^ Coronary PVAT is composed of mature adipocytes and stromal vascular fraction (SVF) cells, the latter of which are closely associated with CHD. For example, macrophages in the periplaque PVAT in coronary arteries were greatly associated with the percentage of arterial obstruction and with the presence of unstable plaques.^11^ Each increase in the M1/M2 macrophage ratio was related to an average 9% increase in arterial obstruction. However, the exact roles of SVF cells in the progression of coronary atherosclerosis remain to be explored.

In the present study, we used single-cell RNA sequencing (scRNA-seq) to (1) depict the cell atlas of coronary PVAT in nonatherosclerosis control (NC) subjects, patients with nonobstructive coronary atherosclerosis (NOCA), and obstructive coronary atherosclerosis (OCA); (2) identify potential pathogenic cell subpopulations in coronary PVAT during progression to atherosclerosis; and (3) identify intercellular signaling changes across conditions and characterize a regulatory network depending on the specific cell type. Our results provide deep insight into atherosclerosis perturbations and provide an essential resource for future drug discovery.

## METHODS

### Data Availability Statement

The data that support the findings of this study are available from the corresponding author upon reasonable request. Sequencing data are available at Genome Sequence Archive (accession number HRA004696), and processed gene expression data are deposited in Gene Expression Omnibus (accession number GSE233870).

### Enrollment of Study Participants

The use of human tissue was approved by the Human Ethics Committee of Fuwai Hospital, Chinese Academy of Medical Sciences. Written informed consent was obtained from each patient. Human heart samples were collected from end-stage heart failure patients who had undergone heart transplantation in the operating room. Coronary PVAT was obtained from PVAT around the left anterior descending artery (LAD) of 50 patients with end-stage heart failure, of whom 10 and 40 were used for scRNA-seq and experimental validation, respectively (Tables S1 and S2). The diagnosis of coronary atherosclerosis and its stage (NOCA or OCA) was based on medical history, symptoms, ECG, laboratory tests, coronary angiography or coronary computed tomography angiography, and coronary hematoxylin and eosin (H&E) staining. In the OCA group, patients had a history of myocardial infarction, angina, and LAD stenosis >70%. And the patients in this group did not experience percutaneous coronary intervention or coronary artery bypass grafting, which might affect the original structure and function of coronary PVAT. In the NOCA group, patients had a plaque in LAD with stenosis <70%. However, these patients did not have a history of myocardial infarction or angina, ST-T change on ECG, or increased values of cardiac enzymes. In the NC group, patients showed no history, symptom, ECG sign, or biochemical sign of CHD, and H&E staining, as well as coronary computed tomography angiography images, showed no lesion in LAD of patients in the NC group (Table S1; Figure S1).

### Tissue Section Preparation

The explanted heart of patients who had undergone heart transplantation was processed using a standardized workflow as we have described previously.^12–14^ The LAD along with the surrounding PVAT was cut transversely at 2-mm intervals. The stenosis severity of 5 LAD segments (proximal, middle, distal, first diagonal, and second diagonal according to the American Heart Association 15-segment model) was preliminarily assessed by 2 experienced pathologists and categorized into 4 degrees (1%–25%, 26%–50%, 51%–75%, and 76%–100%^15^). After this preliminary assessment at the gross pathological level, the LAD segments along with the surrounding PVAT were fixed with formalin for 24 hours, dehydrated, embedded in paraffin, and then serially sectioned into 4-μm-thick section, which were then used for reassessment at the micropathological level by H&E staining.

### H&E Staining

Tissue sections were stained with H&E to determine blood vessel structure. The histological pictures were assessed by 2 pathologists in the Fuwai Hospital. The H&E staining results were used to confirm the assessment of stenosis degree of each LAD segment.

### Tissue Processing and Single-Cell Dissociation

Coronary PVAT, the adipose tissue 5 mm from LAD atherosclerotic site, was collected in 10 mL DMEM (11885084; Gibco) containing 10% fetal bovine serum (10091148; Gibco) on ice. Samples were washed by PBS to remove the remaining blood cells and then cut into small pieces. These small pieces were washed by PBS again and digested in DPBS (Dulbecco’s phosphate-buffered saline; 14040133; Gibco) containing 0.28 U/mL Liberase TM (05401119001; Roche) at 37 °C for 45 minutes, followed by gently shaking. The single-cell suspension was filtered with a 40-µm cell strainer and collected by centrifugation at 400*g* at 4 °C for 5 minutes. The supernatant was discarded, and the cell pellets were resuspended in 1 mL ACK (ammonium-chloride-potassium) lysing buffer (A1049201; Gibco) and incubated for 5 minutes at room temperature. The resulting suspension was further diluted to 10× the initial volume with cold PBS and centrifuged at 400*g* at 4 °C for 5 minutes. The cell precipitate was resuspended in 500 μL cold DMEM containing 10% fetal bovine serum and used immediately.

### Single-Cell Library Preparation and Sequencing

Single-cell suspensions were processed through the 10× Genomics Chromium System (10× Genomics, Pleasanton, CA), followed by the construction of 3′ gene expression v3.1 libraries and sequencing on an Illumina Noveseq6000 sequencer. Briefly, cells (>90% viability), reagents, gel beads, and partitioning oil are loaded onto 10× Chromium Chip G. Ideally, each individual cell is wrapped with an oil drop that contains gel beads and reagents. This creates an independent reaction space for each cell called gel bead in emulsion. The primers provided by the gel bead contain a sequencing primer, barcode, unique molecular identifier (UMI), and poly (dT) sequence. The cell in the gel bead in emulsion is lysed, and incubation of the gel bead in emulsion produces barcoded, full-length cDNA from polyadenylated mRNA. Next, all cDNAs are pooled together, and the steps of general library construction are performed including amplification, fragmentation, end repair, adapter ligation, and sample index polymerase chain reaction (98 °C for 45 s [98 °C for 20 s, 67 °C for 30 s, and 72 °C for 1 minute]×14 cycles; 72 °C for 1 minute). The libraries were then sequenced using Illumina Noveseq6000.

### Data Preprocessing

The raw FASTQ files were aligned to the reference (GRCh38-3.0.0) by Cell Ranger (v6.0.0) and subjected to read filtering, barcode counting, and UMI counting.

### Principal Component Analysis

Pairwise analysis was performed for all 3 groups. The genes used for principal component analysis were selected by pairwise comparison using the Wilcoxon test. The threshold of minimal percentage and logfc are both 0.25. The average expression of differentially expressed genes (DEGs) was used as input for principal component analysis. Principal components were analyzed with prcomp function in stats package with scaling, indicating that the variables were scaled to have unit variance before the analysis takes place.

### Data Quality Control

Doublets were removed with DoubletFinder (details are represented in the next paragraph), and the remaining cells were filtered with nCount_RNA, nFeature_RNA, and percentage of mitochondrial genes. According to reported literature,^16^ the threshold for filtering is median±3 SDs for each variable. This means only cells having nCount_RNA, nFeature_RNA, and percentage of mitochondrial genes less than the determined threshold were subjected to further analysis. Next, cells with UMI detected for HBB or MYL2 were removed. After quality control, 67 936 cells were left.

### Doublet Filtering

DoubletFinder (2.0.3)^17^ was used for doublet prediction. nExp in each data set was calculated with doublet_rate×total_number_of_cell. The doublet rate was estimated with formular total_number_of_cell×0.9/100 000. For 10 000 recovered cells, there would be 9% doublets. The top 10 principal components derived from principal component analysis were used for doublet identification. The proportion of generated artificial doublets in merged real artificial data was set as 0.25. The neighborhood size, defined as the proportion of neighbors in merged data set, was set as 0.02. find.pK was used to evaluate whether the used pK parameter is reasonable.

### Identification of DEGs and Pathway Enrichments

DEGs were identified using Wilcoxon rank-sum test. Upregulated genes in each cell cluster were identified using the FindAllMarkers function with parameters test.use=wilcox, min.pct=0.25, and log_2_fc.threshold=0.25 in Seurat and filtered by *P*<0.05. Genes with a threshold of absolute log_2_fc >0.5 were considered as marker genes for each cluster and subjected to pathway enrichment (gene ontology analysis). The marker genes for each cluster are available in Tables S1 through S6. Gene ontology analysis was performed on DEGs by using an R package ClusterProfiler.^18^ For gene ontology (GO) enrichment, the *P* value cutoff and q value cutoff were set as 0.01 and 0.05, respectively. *P* value was corrected by the Benjamini-Hochberg method.

### Calculation of Single-Cell Scores

Single-cell scores for single-cell data were calculated with the AddModuleScore function in Seurat. The average expression levels of gene list on single-cell level were subtracted by the aggregated expression of control feature sets. All analyzed features are binned based on averaged expression, and the control features are randomly selected from each bin.

### TF Activity Analysis

pySCENIC (0.11.2)^19^ was used to infer potential regulatory TFs (transcription factors) and their target genes in cell clusters. In brief, pySCENIC infers TFs and their target genes from correlations between the expression of genes across cells. A TF and its target genes are defined as a regulon. The regulons are then refined by pruning targets based on enriched motifs. Finally, the activity of a regulon is measured by an AUCell value in each single cell. A high AUCell value indicates high activity and enrichment of a regulon in a cell.

### Cell Cycle Analysis

To predict the cell cycle phases of each individual cell in the given cluster, we used Seurat function CellCycleScoring. The previous well-defined marker genes of S and G2/M phases were used to calculate the S scores and G2/M scores, respectively.^20^

### Trajectory Analysis

Slingshot was used to perform trajectory analysis.^21^ In brief, slingshot first identifies the global lineage structure with a cluster-based minimum spanning tree and then fits simultaneous principal curves to describe each lineage and determines the pseudotime of each cell. To investigate the potential transitions among subclusters, we performed pseudotemporal analysis using the R package Monocle (2.18.0).^22^ Count matrixes were used as inputs, and genes expressed in <10 cells were filtered. Genes expressed in at least 10% cells with *P*<1×10^−10^ and q values <1×10^−10^ in the differential expression testing were defined as ordering genes for trajectory construction. For TNK (T cells and natural killer cells) trajectory, T_2 was set as the root of trajectory, whereas for myeloid trajectory, Ma_4 was set as the root of trajectory.

### Inference and Analysis of Cell-Cell Communication

Intercellular communication network was inferred from single-cell transcriptomic data using CellChat (1.1.0),^23^ which uses mass action model to predict the cell-cell communication probability by integrating gene expression matrix with manually curated databases of signaling ligands, receptors, and cofactors. Pairwise comparisons among NC and atherosclerosis groups were performed to quantitatively predict differential communication pairs and strength. The communication probability on signaling pathway level was determined by summarizing all related ligand/receptor pairs. Difference in overall signaling was tested with paired Wilcoxon test using a *P* value cutoff of 0.05. The calculated communication probability and *P* value of significant ligand/receptor pairs from one cell group to another were visualized as dot plot.

### Multiple Fluorescence Immunohistochemistry

Formaldehyde-fixed paraffin-embedded sections were dewaxed with methanol, subjected to antigen retrieval, blocked for 1 hour, and incubated with primary antibodies overnight at 4 °C. After washing with PBS (10010023; Thermo Fisher, Waltham, MA) 3×, the slides were incubated with appropriate fluorescence-labeled secondary antibodies for 60 minutes. The slides, shielded from light, were washed 3× in PBS and then counterstained and mounted with DAPI (4′,6-diamidino-2-phenylindole; ZSGB-BIO, ZLI-9557). The primary antibodies used were as follows: anti-SPP1 (secreted phosphoprotein 1; ab218237, 4 μg/mL; Abcam), anti-ACTA2 (actin alpha 2; ab7817, 10 μg/mL; Abcam), anti-CD68 (ZM-0060, 10 μg/mL; ZSGB-BIO), anti-SOCS3 (suppressor of cytokine signaling 3; ab280884, 6 μg/mL; Abcam), anti-COL1 (collagen I; ab138492, 8 μg/mL; Abcam), anti-DARC (duffy antigen receptor for chemokines; ab137044, 6 μg/mL; Abcam), anti-CD31 (ZA-0568, 6 μg/mL; ZSGB-BIO), anti-APOE (18254-1-AP, 6 μg/mL; Proteintech), and anti-DCN (decorin; ab268048, 2 μg/mL; Abcam). As described previously, negative controls were performed by substituting a isotype- and concentration-matched normal IgG for the primary antibody,^24,25^ including mouse IgG2a isotype control (ab18415; Abcam) at 10 μg/mL and rabbit IgG isotype control (ab172730; Abcam) at 6 μg/mL. Biotin-bound anti-mouse or anti-rabbit secondary antibodies and streptavidin horseradish peroxidase were used to detect primary antibodies. Antibody staining was visualized using the PerkinElmer Opal Polychromatic IHC System according to the manufacturer’s protocol. The entire slide was scanned using the Vectra Polaris system (PerkinElmer), which initially captured the fluorescence spectra of 5 channels (DAPI, FITC [fluorescein isothiocyanate], Cy3 [cyanine3], Texas red, and Cy5 [cyanine5]).

### Masson Trichrome Staining

Masson trichrome staining was performed using a modified method as we have described previously.^14,26^ The kit used for the staining was purchased from Leagene Biotechnology (DC0033; Beijing, China). Briefly, the sections were deparaffinized, followed by immersion in Bouin solution overnight at room temperature. Then the sections were stained in a Weigert iron hematoxylin solution for 5 minutes and washed in the running water for 5 minutes. After immersion in Biebrich Scarlet-Acid Fuchsin solution for 3 minutes and a brief rinse in distilled water, the slides were incubated in phosphotungstic phosphomolybdic acid solution for 1 minute and 30 s, followed by direct transfer to aniline blue solution, staining for 2 minutes, and a brief rinse in distilled water. After that, the sections were immersed in 1% acetic acid for 2 minutes and then washed using distilled water. Next, the sections were dehydrated through alcohol solutions, cleared in toluene solutions, and mounted with resinous mounting medium. Finally, a Zeiss microscope (Zeiss Axio Observer Z1; Carl Zeiss, Jena, Germany) was used to acquire the images. For quantification, 10 random 20× fields of each independent section were selected. Then, the blue color density of each field was calculated using the ImageJ software as described previously.^27^

### Primary Human Fibroadipogenic Progenitor Cell Isolation and Culture

Coronary PVAT was obtained from 4 patients undergoing heart transplantation and digested as described previously.^28^ Briefly, coronary PVAT was collected in 10 mL DMEM (11885084; Gibco) containing 10% fetal bovine serum (10091148; Gibco) on ice. Samples were washed by PBS to remove the remaining blood cells and then cut into small pieces. These small pieces were washed by PBS again and digested in DPBS (14040133; Gibco) containing 0.1% (w/v) collagenase (LS004174; Worthington) at 37 °C for 60 minutes, followed by gently shaking. The cell suspension was centrifuged at 300*g* at 21 °C for 5 minutes. A vigorous shaking was performed for 10 s to ensure that individual cells are released from the strands of fibrous tissue. Then a second centrifugation (300*g*) was performed and supernatant was discarded, and the cell pellets were resuspended in 10 mL DMEM, followed by a third centrifugation (300*g*). Finally, the supernatant was discarded, and the cell pellets were resuspended in 10 mL fibroblast growth medium (C-23010; Sigma) containing 10% fetal bovine serum and 1% penicillin and streptomycin and culture in 5% CO_2_ at 37 °C. Early passage (P2 and P3) cultures were used for the cell migration assay and cell culture immunostaining.

### Production of Conditioned Medium and Fibroadipogenic Progenitor Cell Migration and Proliferation Assays

PVAT conditioned medium (CM) was harvested from culture human atherosclerotic coronary PVAT after incubation in DMEM for 48 hours. The medium was subjected to filtration using a 0.22-um filter after a 5-minute centrifugation (1200*g*), and the medium was collected for later experiments. Besides, the DMEM-CM was adopted as a control. The effect of PVAT-CM on the migration and proliferation of fibroadipogenic progenitors (FAPs) was analyzed using transwell and EdU (5-ethynyl-2’-deoxyuridine) assay. First, primary FAP cells were plated in equal number for all conditions. Migration assays were performed using 6.5-mm-diameter and 8.0-μm-pore size transwells (3422; Coring) coated with 0.5% gelatin as reported previously.^29^ FAP cells were pretreated with vehicle or anti-CD44 (cluster of differentiation 44) neutralizing monoclonal antibody (10 μg/mL, 15675-1-AP; Proteintech), RGD (arginylglycylaspartic acid) peptide to inhibit integrin receptors (10 μM, A8052; Sigma Aldrich), or both in combination for 30 minutes as described previously.^30^ FAP cells (around 4×10^4^) were added to the upper chamber in migration buffer (medium containing 0.1% BSA). After 6 hours of DMEM-CM or PVAT-CM stimulation at 37 °C, cells were removed from the upper surface of the membranes with a cotton swab, and cells that migrated to the lower surface were fixed with 4% paraformaldehyde for 10 minutes and then stained with 0.1% crystal violet (G1064; Solarbio) for 10 minutes. For cell proliferation assays, 2×10^3^ FAP cells were plated into a 96-well plate. FAP cells were pretreated with vehicle or anti-CD44 neutralizing monoclonal antibody (10 μg/mL), RGD peptide (10 μM), or both in combination for 30 minutes. After 24-hour stimulation of DMEM-CM or PVAT-CM, cells were performed using Cell-Light EdU Apollo594 In Vitro Imaging Kit (C10310-1; Ribobio). Images were obtained by a microscope (Zeiss) at ×10 magnification. For quantification, 4 biological replicates (PVAT-CM from 4 patients and FAP cells from 4 patients) and 5 technical replicates were used.

### Statistical Analysis

Continuous and categorical variables were presented as variables (mean±SD) and frequency (percentages), respectively. DEGs between the cell clusters or DEGs in cell cluster between NC and atherosclerosis were identified using the Mann-Whitney *U* test by the Seurat (version 3.2.0) FindAllMarkers function. For the comparison of cell ratios between each 2 groups, the cell ratios were logit transformed, followed by the Student *t* test (with or without Welch correction) or Mann-Whitney *U* test according to the results of normality test and variance homogeneity test. Comparisons of the cell migration or proliferation between each 2 groups were performed using the Student *t* test. Comparisons of the quantitative real-time polymerase chain reaction result between NC and atherosclerosis were performed using the Student *t* test. For differential expression, proportion, and cell migration and proliferation, *P* values were adjusted for multiple hypothesis testing using the Benjamini-Hochberg method. *P*<0.05 was considered statistically significant. R (version 4.05) was used for the statistical analysis of the scRNA-seq data, and SPSS (version 26.0) was used for the clinical characteristic comparison and Pearson correlation analysis.

A fully detailed description and the Major Resources Table are available in the Supplemental Material.

## RESULTS

### SVF Cell Atlas in Human Coronary PVAT

**Figure 1.**
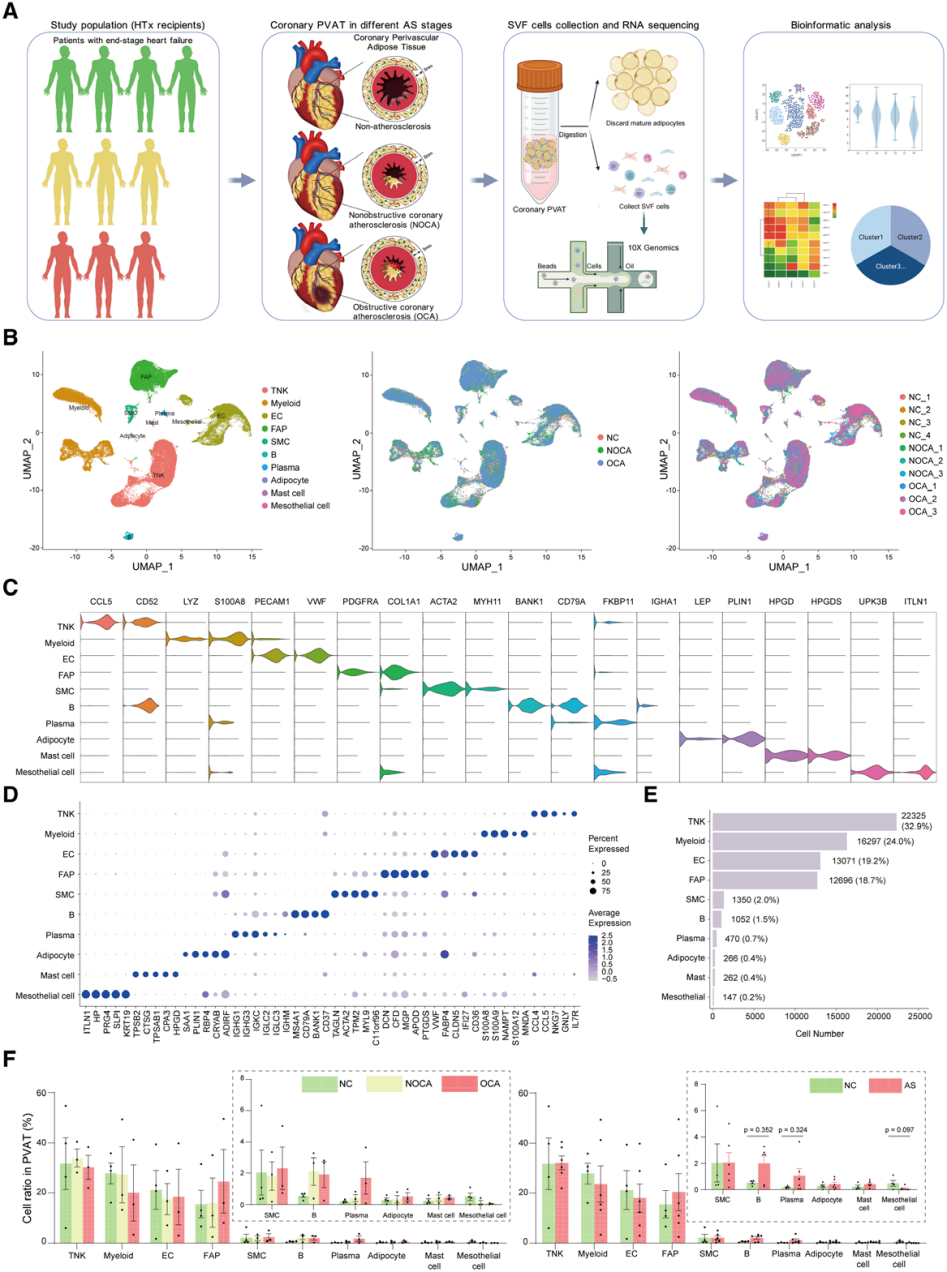
**Stromal vascular fraction (SVF) cell atlas in human coronary perivascular adipose tissue (PVAT). A**, Flowchart of the study including the study groups and single-cell RNA sequencing platform. **B**, Profiles of the Uniform Manifold Approximation and Projection (UMAP) plots of all cells, with each cell color coded for its associated cell type (**left**), the originating group (**middle**), and the corresponding patients (**right**). **C**, Expression of classic marker genes used to define all the cell types. **D**, Dot plot showing the top markers of each cell type. **E**, For each of the cell clusters, the number of cells is shown to provide an overview of all the cells. **F**, Percentage change tendency of each cell type during the progression of atherosclerosis (AS). The Mann-Whitney *U* test (for B cells and mesothelial cells) or Student *t* test with Welch correction (for plasma cells) was performed to compare the log-transformed proportion of cell subpopulations between each 2 groups; *P* values were adjusted for multiple hypothesis testing using the Benjamini-Hochberg method. EC indicates endothelial cell; FAP, fibroadipogenic progenitor; HTx, heart transplantation; NC, nonatherosclerosis control; NK, natural killer; NOCA, nonobstructive coronary atherosclerosis; OCA, obstructive coronary atherosclerosis; and SMC, smooth muscle cell.

In the present study, we aimed to comprehensively examine the cell composition of the SVF of coronary PVAT. A total of 10 human coronary PVAT samples were collected, including 4 from the NC group, 3 from the NOCA group, and 3 from the OCA group (Figure 1A). Patients were allocated to their respective group based on the clinical history, imaging information, and histological characterization of the corresponding coronary artery (Tables S1 and S2; Figure S1). Freshly collected coronary PVAT was digested enzymatically to prepare the single-cell suspension, followed by scRNA-seq using the 10× Genomics Chromium platform. Then, the sequencing reads were demultiplexed, mapped to the GRCh38 human genome, and counted by unique molecular identifier. Next, quality control was performed, leaving 67 936 high-quality cells for further analysis (Table S3; Figure S2). Additionally, many long noncoding RNAs and microRNAs were detected (Table S4). Those results suggested that scRNA-seq captured full-length transcripts comprehensively.

We clustered the cells and visualized the results using unsupervised clustering and Uniform Manifold Approximation and Projection, respectively. Cell type annotation is a crucial step in analyzing the scRNA-seq data set; tools have been developed for automatic cell type identification,^31^ yet manual annotation is necessary to avoid mistaken. In the study, we combined SingleR with manual annotation to assign cell types to the different clusters. A total of 25 subclusters were obtained, and then 10 major cell types were identified (Figure S3; Table S5). Each of the major cell types came from 10 patients (Figure 1B). Based on the canonical markers, we defined the 10 clusters as T cells and natural killer (NK) cells, myeloid cells, endothelial cells (ECs), FAP cells, smooth muscle cells (SMCs), B cells, plasma cells, adipocytes, mast cells, and mesothelial cells (Figure 1C; Figure S3). The above 10 clusters showed specific gene expression patterns and transcripts (Figure 1D). The marker genes for each major cluster are available in Supplemental Excel File I. The T/NK cell cluster was the largest population, followed by the myeloid cell, EC, and FAP cell clusters (Figure 1E). No statistical difference of cellular proportion was found among groups (Figure 1F).

### CD4^+^ T Cells Showed Anti-Inflammatory Function in the Human Nonatherosclerotic Coronary PVAT

**Figure 2.**
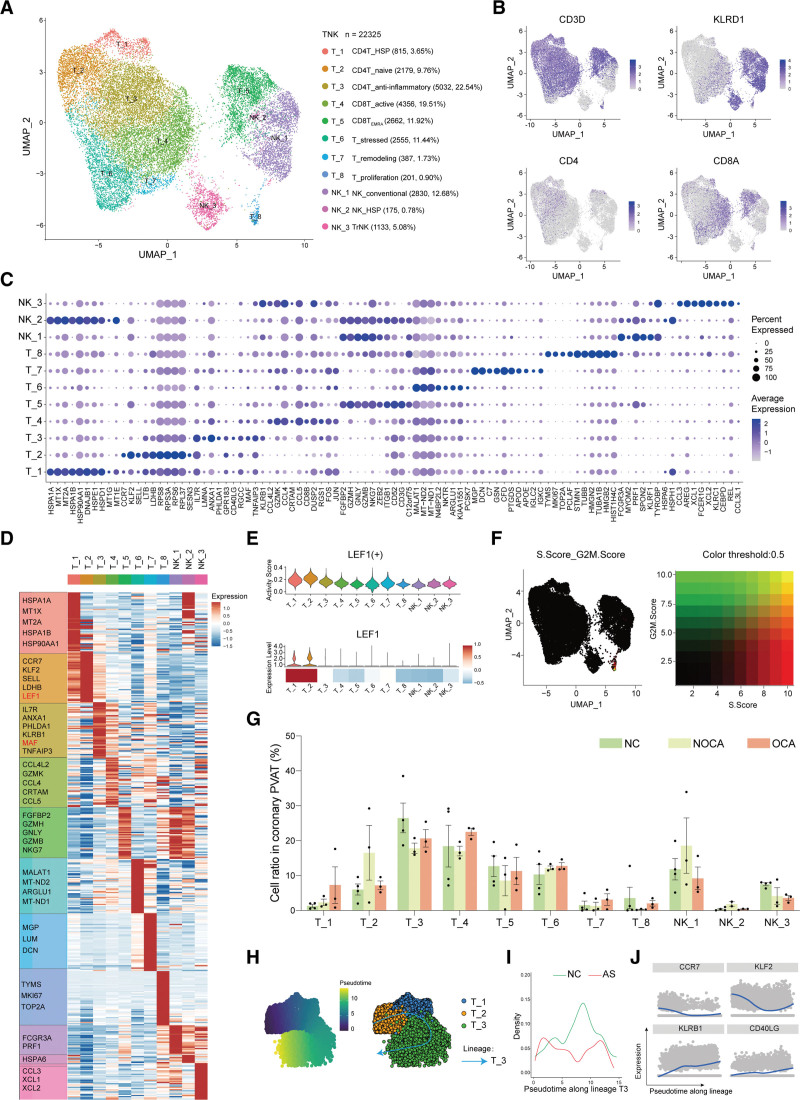
**An anti-inflammatory CD4^+^ T-cell subpopulation decreased in the human atherosclerotic coronary perivascular adipose tissue (PVAT). A**, A Uniform Manifold Approximation and Projection (UMAP) plot of all T/natural killer (NK) cells colored according to cluster. **B**, Relative expression of classical markers in T/NK cells. **C**, Dot plot showing the top 10 marker genes of each T/NK subcluster. Dot color and size correspond to the expression of each gene and the proportion of cells expressing each gene, respectively. **D**, Mean expression of top marker genes of each T/NK subcluster. TF (transcription factor) genes are colored in red. **E**, The expression level and regulon activity of TF in T/NK subclusters. **F**, UMAP showing the phase S score and phase G2/M score of every cell. **G**, The ratio of each T/NK subcluster in the different phases. **H** through **J**, Trajectory analysis of T1, T2, and T3 using Slingshot. AS indicates atherosclerosis; NC, nonatherosclerosis control; NOCA, nonobstructive coronary atherosclerosis; and OCA, obstructive coronary atherosclerosis.

A total of 22 325 T/NK cells were further divided into 11 subclusters of CD4^+^ T cells (*CD4*)/CD8^+^ T cells (*CD8*)/NK cells (*KLRD1*; Figure 2A and 2B).

CD4^+^ T cells consisted of T_1, T_2, and T_3 (Figure 2A and 2B). The T_1 subcluster was defined as CD4T_HSP due to the high expression of *HSP* (heat shock protein) genes. T_1 also highly expressed metalloprotease genes like *MT1X* and *MT2A* (Figure 2C and 2D). The T_2 subcluster was assigned as naive CD4^+^ T cells (CD4T_navie) with high expression of naive gene or proliferation markers, including *CCR7*, *KLF2*, *SELL*, *LDHB*, and *LEF1* (Figure 2D). We identified a potential transcriptional factor *LEF1* that might be responsible for differentiation of T_2 using the single-cell regulatory network inference and clustering analysis (Figure 2E). The T_3 subcluster was categorized as anti-inflammatory CD4^+^ T cells (CD4T_anti-inflammatory) due to the high expression of anti-inflammatory or inflammation-regulatory genes *IL7R*,^32^ *ANXA1*,^33^ *PHLDA1*,^34^ *KLRB1*,^35^ *MAF*,^36^ and *TNFAIP3*^37^ (Figure 2D), and the functions of negative regulation of immune system process, positive regulation of anti-inflammatory cytokine IL (interleukin)-2 (Figure S4A).

The CD8^+^ T cells included T_4 and T_5. The T_4 subcluster was defined as active CD8^+^ T cells (CD8T_active) with high expression of the proinflammatory genes *CCL4L2*, *CCL4*, and *CCL5*, as well as the cytotoxic genes *GZMK* and *CRTAM*. T_5 was assigned as terminally differentiated effector CD8^+^ T cells (CD8T_EMRA_) because this subcluster demonstrated high expression of the cytotoxicity-associated genes *FGFBP2*, *GZMH*, *GNLY*, *GZMB*, and *NKG7* (Figure 2D).

T_6, T_7, and T_8 mainly contained CD8^+^ T cells, with few CD4^+^ T cells (Figure 2B). The subcluster of T_6 was renamed as T_stressed because these cells expressed high levels of stress-related genes (*MALAT1*^38^ and *ARGLU1*^39^) and mitochondrial genes. T_7 was categorized as T_remodeling because it expressed the fibroblast genes *MGP*, *LUM*, and *DCN* (Figure 2D). T_8 was defined as T_proliferation because it demonstrated high expression of the proliferation-related genes *TYMS* and *MKI67* (Figure 2D). The proliferative state of T_8 was also confirmed by the cell cycle analysis (Figure 2F).

NK_1 was classified as conventional NK (NK_conventional) because they highly expressed cytotoxic genes *FCGR3A*, *FGFBP2*, and *PRF1*. NK_3 was defined as tissue resident NK since the high expression of *CCL3*, *XCL1*, and *XCL2* (Figure 2D). Our study confirmed their presence in the human coronary PVAT. Besides, there was a small number of NK_2 (NK_HSP), which expressed high levels of *HSP* genes. The marker genes for each TNK subcluster are available in Supplemental Excel File II.

Interestingly, the cell ratio of the 3 CD4^+^ T-cell subclusters (T_1, T_2, and T_3) was increased in OCA, NOCA, and NC, respectively (Figure 2G). To investigate the developmental pathways of the 3 CD4^+^ T-cell subclusters, we performed Slingshot^21^ and Monocle2^40^ analyses (Figure 2H through 2J; Figure S4B). For Slingshot analysis, T_2 was determined as the root because it highly expressed naive markers (Figure 2C). T_2 subsequently evolved into T_1 and T_3 (Figure 2H), and the cell density plot shows that NC was enriched in the second half of lineage T_3 compared with atherosclerosis (Figure 2I). Along the T_3 lineage, the expression level of naive markers (*CCR7* and *KLF2*) decreased, while the expression level of anti-inflammatory markers (*KLRB1* and *CD40LG*) increased (Figure 2J). Similarly, we examined the distributions of CD4^+^ T cells along pseudotime separately for atherosclerosis and NC using Monocle2. The CD4^+^ T cells of the NC were clearly shifted toward more differentiated states compared with those of the atherosclerosis: most of the T cells in the NC were placed in the latest pseudotime corresponding to anti-inflammatory T cells (Figure S4B). Furthermore, anti-inflammatory/inflammation-regulatory genes (like *IL7R*, *ANXA1*, and *KLRB1*; Figure S4C) and pathways (like regulation of IL-2 production; Figure S4D) were decreased in CD4^+^ T cells (T_1, T_2, and T_3) from atherosclerosis compared with those from NC. Overall, these results confirmed that CD4^+^ T cells in NC exhibited anti-inflammatory function, while in atherosclerosis, the anti-inflammatory function was drastically reduced.

### Identification of SPP1^+^ Macrophages in Human PVAT Surrounding the Atherosclerotic Coronary Artery

**Figure 3.**
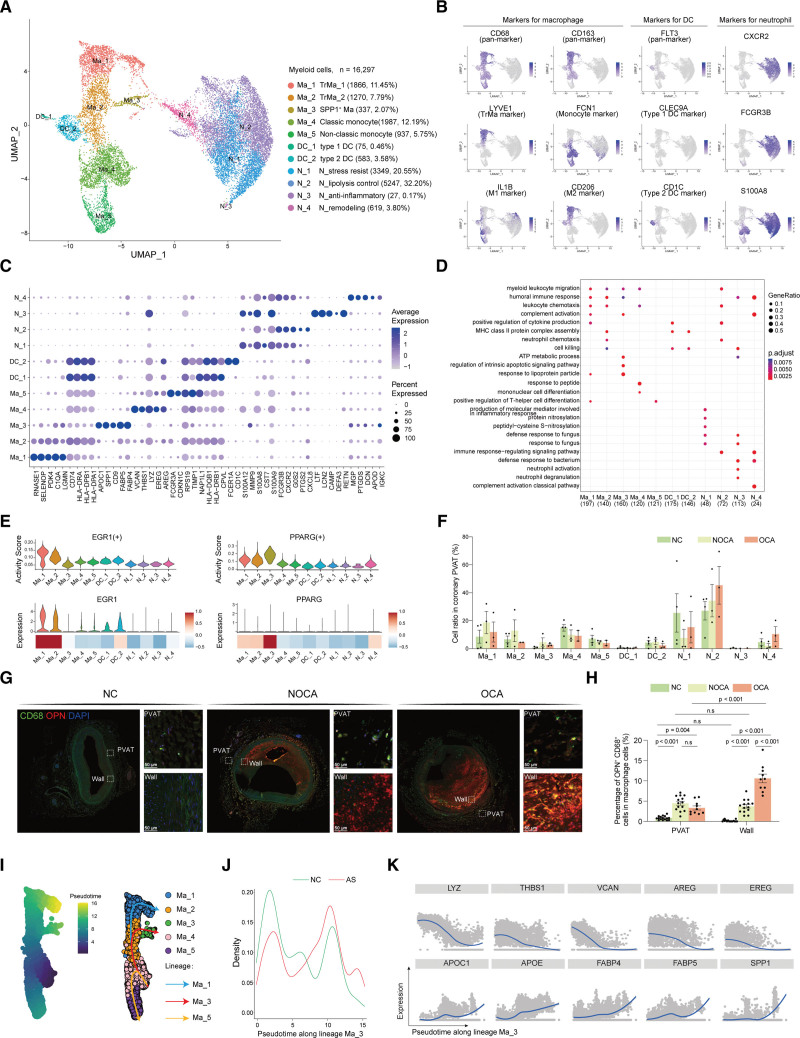
**Identification of *SPP1*^+^ (secreted phosphoprotein 1) macrophages in human perivascular adipose tissue (PVAT) surrounding atherosclerotic coronary artery. A**, A Uniform Manifold Approximation and Projection (UMAP) plot of all myeloid cells colored according to cluster. **B**, Relative expression of classical markers in myeloid cells. **C**, Dot plot showing the top 5 marker genes of each myeloid subcluster. Dot color and size correspond to the expression of each gene and the proportion of cells expressing each gene, respectively. **D**, The top 5 enriched gene ontology biological process terms of each myeloid subcluster. **E**, The expression level and regulon activity of TFs (transcription factors) in myeloid subpopulations. **F**, The ratio of each myeloid subcluster in the different phases. **G**, Multiple labeling staining for *SPP1*^+^ macrophage. Scale bar, 100 µm. **H**, Quantification of (**G**) cell ratio per image (n=16 in nonatherosclerosis control [NC], n=14 in nonobstructive coronary atherosclerosis [NOCA], and n=10 in obstructive coronary atherosclerosis [OCA]). Mann-Whitney *U* test was performed to compare the cellular ratio between each 2 groups; *P* values were adjusted for multiple hypothesis testing using the Benjamini-Hochberg method. **I** through **K**, Trajectory analysis of Ma_1, Ma_2, Ma_3, Ma_4, and Ma_5 using Slingshot. AS indicates atherosclerosis; COL, collagen; and OPN, osteopontin.

In total, 16 297 myeloid cells were clustered into 11 types of macrophages (*CD68* and *CD163*)/dendritic cells (DCs; *FLT3*)/neutrophils (*CXCR2*, *FCGR3B*, and *S100A8*; Figure 3A and 3B; Figure S5A).

Macrophages were divided into 5 subclusters (Ma_1 to Ma_5). Ma_1 and Ma_2 were tissue-resident macrophages because these 2 clusters expressed high levels of tissue-resident macrophage marker *LYVE1*^41^ (Figure 3B), which was further confirmed by the negative expression of *CCR2* (Figure S5B). Ma_3 was defined as *SPP1*^+^ Ma due to its marker gene *SPP1*, and Ma_4 and Ma_5 were monocyte-derived macrophages due to their expression of monocyte marker gene *FCN1* (Figure 3B and 3C). The Ma_1 cluster also expressed genes *RNASE1*^42^ and *SELENOP*,^43^ indicating the antioxidant role of the Ma_1 cluster. Ma_2 shared similar marker genes with Ma_1. In addition, Ma_2 expressed major histocompatibility complex class II molecules, which specifically present antigens to CD4 T lymphocytes^44^ (Figure 3C and 3D). Of note, the minor cluster Ma_3 (*SPP1*^+^ Ma) highly expressed several lipid metabolism–related genes, including lipid influx transporters *FABP4* and *FABP5*, lipid binding and transport gene *APOC1*, lipid efflux transporter *APOE*, and lipid metabolism regulator and cytokine *SPP1* (Figure 3C and 3D; Figure S5C), which made the cells in this cluster similar to the previously reported lipid-associated macrophages.^45,46^ The Ma_4 was a classic monocyte because the cluster highly expressed the epidermal growth factor–encoding genes *EREG* and *AREG*, the matrix glycoprotein-encoding gene *THBS1*, and the chondroitin sulfate proteoglycan-encoding gene *VCAN*. In contrast, the Ma_5 expressed nonclassical monocyte markers *FCGR3A*,^47^ *SPN*, and *SIGLEC10* and was, therefore, defined as a nonclassic monocyte (Figure 3C; Figure S5C).

The DC cluster expressed high levels of major histocompatibility complex genes, which is in agreement with the identity of DCs as professional antigen-presenting cells. The DC_1 cluster highly expressed markers of classical type 1 DC, including *CLEC9A* and *IDO1*. The DC_2 cells expressed markers of classical type 2 DC, including *CD1C*, *FCER1A*, and *CLEC10A* (Figure 3B and 3C; Figure S5C).

The neutrophil cluster N_1 expressed *CST7*, which is a cysteine peptidase inhibitor known to be expressed in NK and CD8^+^ T cells during steady-state conditions.^48^ N_1 (N_stress resist) also expressed *TXN*, which helps NK cells to acquire resistance against oxidative stress.^49^ The N_2 (N_lipolysis control) cluster highly expressed the genes encoding G0S2, suggesting a role in lipolysis control. The N_3 (N_anti-inflammatory) cluster expressed *LTF*, *CD24*, and *MMP8*, indicating an anti-inflammatory and profibrotic role for N_3. N_4 (N_remodeling) demonstrated high expression of *CFD* and *DCN*, indicating a fibroblast-like cell type.The marker genes for each myeloid subcluster are available in Supplemental Excel File III.

The single-cell regulatory network inference and clustering analysis identified *EGR1* as a candidate TF underlying the differences in gene expression in the Ma_1 cluster (Figure 3E). It has been reported that *EGR1* has proatherogenic function in atherosclerosis.^50^ Adipogenic marker gene *PPARG*^51^ was identified as candidate TF underlying the differences in gene expression in the Ma_3 cluster (Figure 3E), indicating the adipogenic potential of Ma_3.

After defining each myeloid subcluster, we compared the cell ratio of each subcluster among 3 groups and found that Ma_3 was accumulated in atherosclerosis compared with NC (Figure 3F; Figure S5D), which was further validated by counting OPN (osteopontin; encoding gene *SPP1*) and CD68-positive cells using pathological staining pictures (Figure 3G). The results showed that compared with NC, the proportion of OPN^+^ macrophages in PVAT of NOCA and OCA increased by 5.42-fold and 4.04-fold, respectively (Figure 3H), while there was no significance between OCA and NOCA (Figure 3H). To further analyze the pseudotime trajectory of Ma_3, we performed the trajectory analysis using Slingshot and Monocle2. As the results of Slingshot show in Figure 3I, Ma_4 (classic monocyte) was set as the root as described previously,^52^ and 3 distinct trajectories were observed: Ma_4 branched into 2 different lineages to form Ma_5 (Ma_5 lineage) and Ma_2, the latter of which further branched into the Ma_1 and Ma_3 lineages (Figure 3I). Density plots revealed that cells in atherosclerosis were enriched in the end of Ma_3 lineage, confirming our above observations of Ma_3 enrichment in atherosclerosis (Figure 3J). Along the Ma_3 lineage, the expression level of classic monocyte markers (*LYZ*, *THBS1*, *VCAN*, *AREG*, and *EREG*) decreased, while the expression level of Ma_3 markers (*APOC1*, *APOE*, *FABP4*, *FABP5*, and *SPP1*) increased (Figure 3K). Consistently, the results of Monocle2 showed that Ma_4 was the root of trajectory and it was more likely to differentiate into Ma_3 in atherosclerosis compared with NC (Figure S5E).

### Atherosclerosis Was Accompanied With the Activation of EC and SMC Subclusters in Coronary PVAT

**Figure 4.**
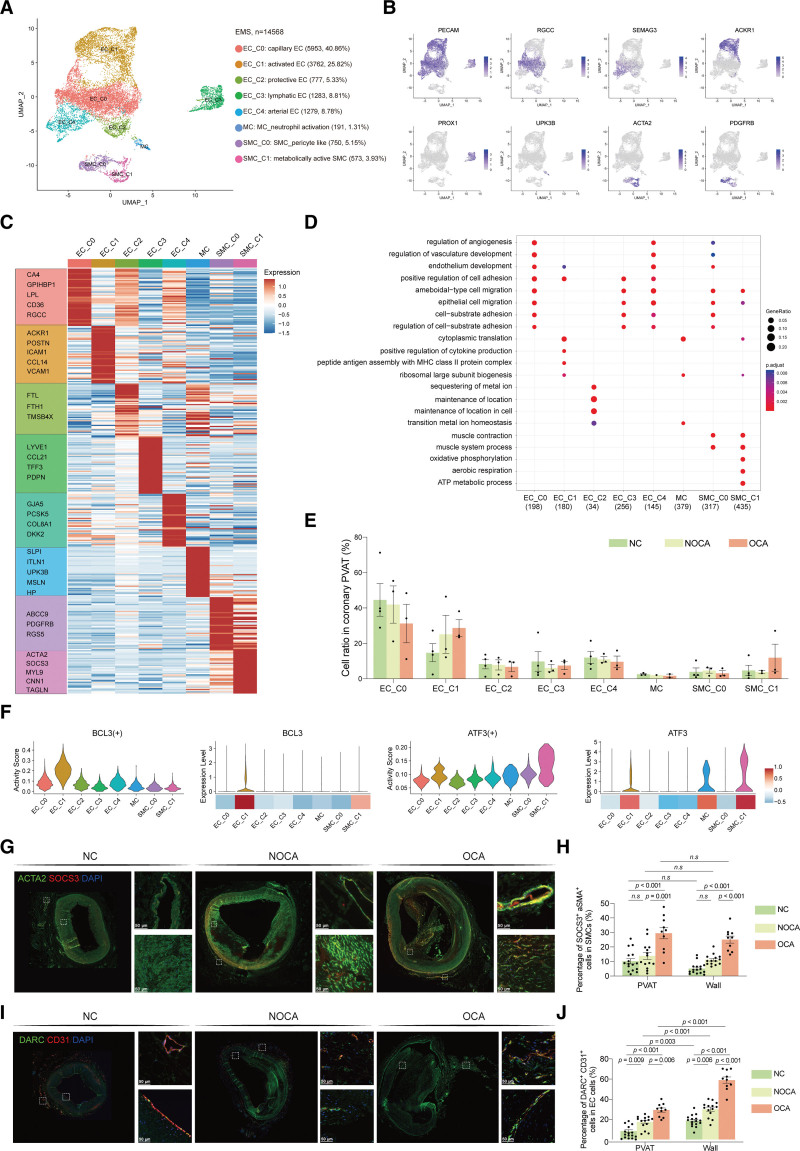
**Atherosclerosis was accompanied with the activation of endothelial cell (EC) and smooth muscle cell (SMC) subclusters in coronary perivascular adipose tissue (PVAT). A**, A Uniform Manifold Approximation and Projection (UMAP) plot of all ECs, mesothelial cells, and SMCs (EMSs) colored according to cluster. **B**, Relative expression of classical markers in EMS cells. **C**, Mean expression of top marker genes in EMS clusters. **D**, The top 5 enriched gene ontology biological process terms of each EMS subcluster. **E**, The ratio of each EMS subcluster in the different phases. **F**, The expression level and regulon activity of TFs (transcription factors) in EMS subpopulations. **G**, Immunofluorescence staining of SOCS3 (red) and ACTA2 (green) in coronary PVAT in different phases. Scale bar, 100 µm. **H**, Quantification of (**G**) cell ratio per image (n=16 in nonatherosclerosis control [NC], n=14 in nonobstructive coronary atherosclerosis [NOCA], and n=10 in obstructive coronary atherosclerosis [OCA]). **I**, Immunofluorescence staining of DARC (red) and CD31 (green) in coronary PVAT in different phases. Scale bar, 100 µm. **J**, Quantification of (**I**) cell ratio per image (n=16 in NC, n=14 in NOCA, and n=10 in OCA). Mann-Whitney *U* test was performed to compare the cellular ratio between each 2 groups; *P* values were adjusted for multiple hypothesis testing using the Benjamini-Hochberg method. ACTA2 indicates actin alpha 2; DARC, duffy antigen receptor for chemokines; MC, mesothelial cell; and SOCS3, suppressor of cytokine signaling 3.

The EC, mesothelial cell, and SMC clusters formed the third population of the SVF. A total of 14 568 ECs, mesothelial cells, and SMCs could be further spilt into 8 types of ECs (*PECAM1*)/mesothelial cells (*UPK3B*)/SMCs (*ACTA2* and *PDGFB*; Figure 4A and 4B). Each cluster expressed specific enrichment marker genes (Figure 4C).

We identified 5 clusters of ECs, namely EC_C0 to EC_C4 (Figure 4A). EC_C0 expressed high levels of the genes encoding RGCC (regulator of cell cycle), CA4 (carbonic anhydrase 4), and GPIHBP1 (glycosylphosphatidylinositol-anchored high-density lipoprotein binding protein 1) and was annotated as capillary EC.^53^ This cluster also highly expressed the genes encoding LPL (lipoprotein lipase) and CD36, which are expressed in microvascular ECs from adipose tissue and are involved in fatty acid transport (Figure 4C).^54^ The EC_C1 cluster specifically expressed leukocyte recruitment-related gene *ACKR1* (encoding protein DARC)^55^ and profibrotic gene *POSTN* (Figure 4C).^56^ The GO analysis also suggested the proinflammatory role of EC_C1 (Figure 4D). Thus, EC_C1 was annotated as activated ECs. The EC_C2 highly expressed *FTL* and *FTH1*, both of which could defend against pathogens by regulating iron availability.^57^ The EC_C2 also expressed *TMSB4X*, which could promote cell survival and inhibit cell apoptosis (Figure 4C).^58^ Therefore, EC_C2 was annotated as protective ECs. EC_C3 was defined as lymphatic EC because of the high expression of *PROX1*, *LYVE1*, and *CCL21.* The EC_C4 cluster expressed the genes encoding SEMA3G (semaphorin 3G) and GJA5 (gap junction protein alpha 5), the latter of which has functional importance in flow-driven arterial remodeling and collateral arterial network development.^59^ Therefore, the EC_C4 cluster was identified as arterial ECs. Mesothelial cells (MC_neutrophil activation) highly expressed *UPK3B*, *C3*, *HP*, and *SLPI*, suggesting a role in neutrophil activation and degranulation (Figure 4C and 4D).

SMCs were characterized by their expression of *ACTA2* (Figure 4B). The SMC_C0 (SMC_pericyte like) cluster expressed the pericyte marker *ABCC9*^53^ and the proliferation-associated *RGS5*.^60^ SMC_C1 cells expressed considerably higher levels of *SCOS3*, *CNN1*, *ACTA2*, and *TAGLN*, indicating a mature and arterial origin. SMC_C1 was highly activated in ATP synthesis; therefore, we defined SMC_C1 as a metabolically active SMC (Figure 4C and 4D). The marker genes for each EC, mesothelial cell, and SMC subcluster are available in Supplemental Excel File IV.

Cellular proportion analysis revealed an increase of EC_C1 in the atherosclerosis group compared with the NC group and SMC_C1 in the OCA group compared with the other 2 groups (Figure 4E). Interestingly, both the EC_C1 and SMC_C1 are activated clusters based on the above annotations. The activated state of EC_C1 and SMC_C1 was associated with TF regulating cell migration (like *BCL3*^61^) and inflammation (like *ATF3*^62^), respectively (Figure 4F). The accumulation of SMC_C1 (SOCS3^+^ ACTA2^+^) in the group of OCA (Figure 4G and 4H; Figure S6) and EC_C1 (DARC^+^ CD31^+^) in the group of atherosclerosis (Figure 4I and 4J; Figure S6) was further validated using pathological staining. The results showed that compared with NC, the proportion of SOCS3^+^ SMCs in PVAT of OCA increased by 2.89-fold (Figure 4H), and the proportion of DARC^+^ ECs in PVAT of NOCA and OCA increased by 2.1-fold and 3.51-fold, respectively (Figure 4J).

### Profibrotic FAP Subclusters Expanded in PVAT Surrounding the Atherosclerotic Coronary Artery

**Figure 5.**
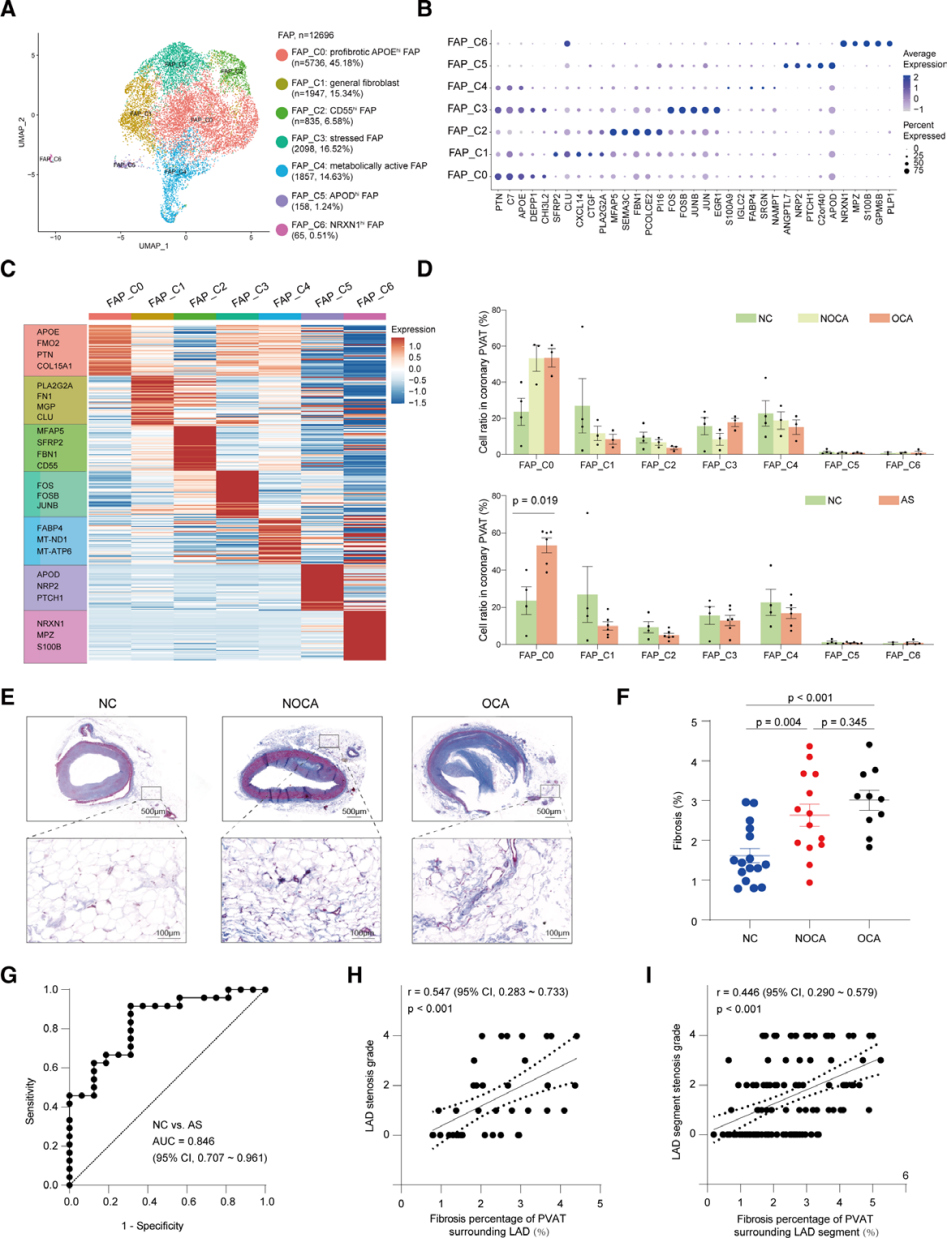
**Profibrotic fibroadipogenic progenitor (FAP) subclusters expanded in perivascular adipose tissue (PVAT) surrounding atherosclerotic coronary artery. A**, A Uniform Manifold Approximation and Projection (UMAP) plot of all FAP cells colored according to cluster. **B**, Dot plot showing the top 5 marker genes of each FAP subcluster. Dot color and size correspond to the expression of each gene and the proportion of cells expressing each gene, respectively. **C**, Mean expression of top marker genes in FAP clusters. **D**, The ratio of each FAP subcluster in the different phases. The Mann-Whitney *U* test for FAP_C0 cells was performed to compare the log-transformed proportion of cell subpopulations between nonatherosclerosis control (NC) and atherosclerosis (AS). For differential cellular proportion, *P* values were adjusted for multiple hypothesis testing using the Benjamini-Hochberg method. **E**, Masson staining of PVAT from different phases. **F**, Quantification of (**E**) fibrosis percentage per image (n=16 in NC, n=14 in nonobstructive coronary atherosclerosis [NOCA], and n=10 in obstructive coronary atherosclerosis [OCA]). The Mann-Whitney *U* test was performed to compare the log-transformed proportion of cell subpopulations between each 2 groups. Scale bar, 500 µm (**top**) or 100 µm (**bottom**). **G**, Receiver operating characteristic (ROC) curves for PVAT fibrosis to distinguish NC from AS. **H** and **I**, The correlation between fibrosis percentage of PVAT surrounding left anterior descending artery (LAD) and LAD stenosis grade based on vessel (**H**) and segment (**I**) evaluation. AS indicates atherosclerosis; and FAP; fibroadipogenic progenitor.

A total of 12 696 FAP cells could be further spilt into 7 types (Figure 5A and 5B; Figure S7A). All of these 7 subclusters expressed positive markers for cultured adipose-derived stromal/stem/progenitor cell (like *PDGFRA*, *CD34*, *CD59*, or *ITGB1*) and lacked the expression of adipose-derived stromal/stem/progenitor cell negative markers like *CD45*, *CD14*, *CD19*, and *CD11b* (Figure S7B). FAP_C0 demonstrated high expression of adipocyte progenitor cell marker APOE^54^ (Figure 5B and 5C). FAP_C0 also highly expressed profibrotic genes, such as *PTN*, *COL15A1*, and *PTGDS* (Figure 5C). The GO analysis further confirmed the ECM (extracellular matrix) organization function of FAP_C0 (Figure S7C and S7D). Therefore, FAP_C0 was annotated as profibrotic APOE^hi^ FAP. FAP_C1 was identified as general fibroblast as the expression of *PLA2G2A* and *FN1* (Figure 5B and 5C). FAP_C2 highly expressed adipose stem cell marker gene *CD55* and was, therefore, defined as CD55^hi^ adipose stem cell (Figure 5C). FAP_C3 was described as stressed FAP because it expressed high levels of stress response genes *JUN*, *FOS*, and *JUNB*, as well as *HSP* genes (Figure 5C). FAP_C4, annotated as metabolically active FAP, highly expressed *FABP4* and *MT-ATP6* (Figure 5C). FAP_C5 and FAP_C6 were annotated as APOD^hi^ FAP and NRXN1^hi^ FAP as the expression of *APOD* and *NRXN1*, respectively (Figure 5B and 5C). The marker genes for each FAP subcluster are available in Supplemental Excel File V.

Then we compared the cell ratio of each FAP subcluster among 3 groups and found the ratio of FAP_C0 was increased in atherosclerosis compared with NC (Figure 5D). Immunostaining further confirmed a 1.93-fold and 2.44-fold increase of FAP_C0 (APOE^+^ DCN^+^) cells in NOCA and OCA compared with NC, respectively (Figure S7E and S7F). Because FAP_C0 played a profibrotic role, we further examine the fibrosis percentage of coronary PVAT among different stages of coronary atherosclerosis, and results showed the fibrotic remodeling degree of coronary PVAT was significantly increased by 1.63-fold and 1.86-fold in NOCA and OCA compared with NC, respectively (Figure 5E and 5F). The PVAT fibrosis degree could effectively distinguish atherosclerosis from NC, with the area under the curve of 0.846 (Figure 5G). The conclusion was further examined by selected ECM genes and ECM-related GOBP (gene ontology biological process) terms using scRNA-seq data, as well as quantitative real-time polymerase chain reaction (Figure S7G through S7I). To explore whether the fibrotic remodeling phenotype of coronary PVAT was associated with the degree of coronary stenosis, we performed Pearson correlation analysis and found the fibrotic remodeling level in PVAT surrounding LAD was positively related to the LAD stenosis degree at the level of both vessel and segment (r>0.44; *P*<0.001; Figure 5H and 5I). These results revealed the fibrotic remodeling of coronary PVAT as an obvious phenotype of coronary atherosclerosis, and the expansion of profibrotic FAP subclusters might be the potential pathogenesis.

### Cell-Cell Interaction Analysis Unveiled the SPP1^+^ Macrophage Promoted the Fibrotic Remodeling of FAP in Coronary PVAT

**Figure 6.**
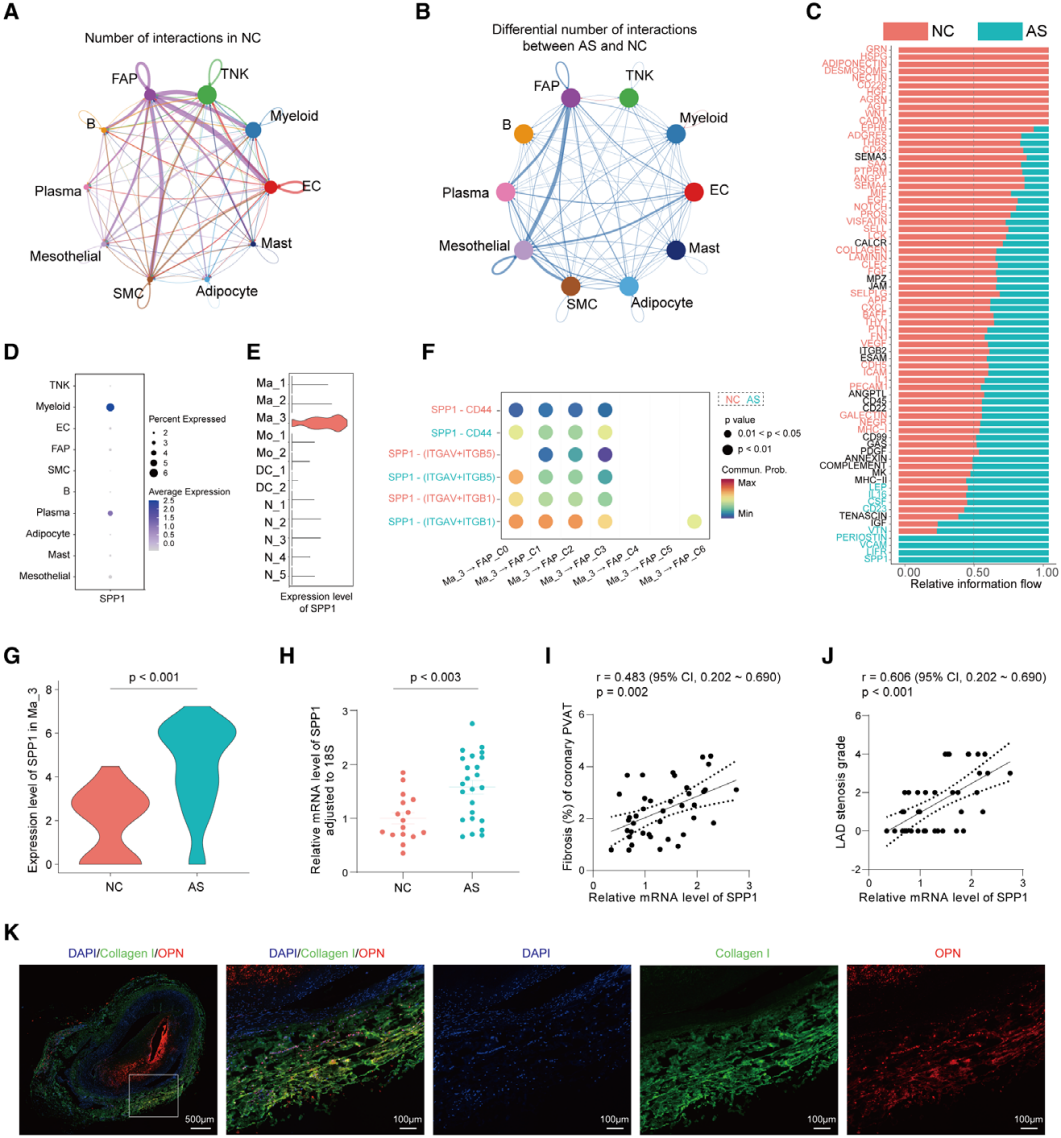
**Cell-cell interaction analysis unveiled the *SPP1*^*+*^ (secreted phosphoprotein 1) macrophage promoted the fibrotic remodeling of fibroadipogenic progenitor (FAP) in coronary perivascular adipose tissue (PVAT). A**, Net plot showing the interaction number among the 10 major cell clusters in nonatherosclerosis control (NC). Each dot indicates 1 cell cluster, and its size is proportional to the number of cells in the cluster. The thickness of the lines connecting cell clusters indicates the interaction number. The color of connecting lines is as same as the color of the source cell cluster. The number on each connecting line indicates the quantity of significant ligand-receptor pairs between any 2 pairs of cell clusters. The loops indicate autocrine circuits. **B**, Overall intercellular communication between each pair of cell populations in the comparison of NC and atherosclerosis (AS). Each dot indicates 1 cell cluster, and its size is proportional to the number of cells in the cluster. The thickness of the lines connecting cell clusters indicates the differential interaction number (blue line indicates an increase in NC, and red line indicates an increase in AS). **C**, Significant signaling pathways were ranked based on differences in the overall information flow within the inferred networks between NC and AS. **D**, Dot plot showing the expression of SPP1 among all the 10 cell clusters. **E**, Violin plot showing the expression of SPP1 among all the 11 myeloid cell subclusters. **F**, The ligand-receptor pairs showing significant probabilities in specificity between Ma_3 and FAP subclusters. **G**, Violin plot showing the expression of SPP1 among Ma_3 in the comparison of NC and AS. Differentially expressed genes (DEGs) in cell cluster between NC and AS were identified using the Mann-Whitney *U* test; *P* values were adjusted for multiple hypothesis testing using the Benjamini-Hochberg method. **H**, Relative mRNA level of SPP1 in the coronary PVAT from NC or AS. Comparisons of the quantitative polymerase chain reaction (qPCR) result between NC and AS were performed using the Student *t* test. **I** and **J**, The correlation between SPP1 mRNA of PVAT surrounding left anterior descending artery (LAD) and PVAT fibrosis (**I**) or LAD stenosis grade (**J**) evaluation. **K**, Immunofluorescence staining of OPN (osteopontin; red) and COL1 (collagen I; green) in coronary PVAT in AS. Scale bar, 100 to 500 µm. AS indicates atherosclerosis; EC, endothelial cell; FAP, fibroadipogenic progenitor; MC, mesothelial cell; NK, natural killer; and SMC, smooth muscle cell.

To investigate the cell-cell interaction network among the cell types identified in our present work, we performed cell-cell interaction analysis using Cellchat.^23^ In PVAT surrounding nonatherosclerotic coronary arteries, FAP played a central role in cellular cross talk because it showed the highest count of interactions with other cell types (Figure 6A). However, such interactions were decreased in PVAT surrounding atherosclerotic coronary arteries (Figure 6B). We further explored the differential cell-cell communication pathways between NC and atherosclerosis and found PVAT in atherosclerosis showed unique pathways related to fibrosis, like *SPP1*,^63^ *VCAM*,^64^ and *POSTN*^65^ (Figure 6C). Of note, *SPP1* was mainly expressed in myeloid cells, especially in Ma_3 (Figure 6D and 6E). Considering FAP was the main fibrosis-producing cell type among the cellular population in PVAT, we thus performed the receptor-ligand pair analysis between Ma_3 and 7 FAP subclusters and identified several enhanced interactions like SPP1-CD44 and SPP1-integrin (ITGAV [integrin subunit alpha V], ITGB5 [integrin subunit beta 5], and ITGB1 [integrin subunit beta 1]) in atherosclerosis (Figure 6F). The SPP1 signaling between Ma_3 and all subclusters, as well as between all subclusters and FAP, was further analyzed by Cellchat to further determine the relative effects of Ma_3 on FAP (Figure S7). Results showed that SPP1-CD44 interaction existed between Ma_3 and several subclusters (including FAP subclusters) while SPP1-integrin interactions specifically existed between Ma_3 and FAP subclusters (Figure S8A). Similarly, the SPP1 signaling on FAP was mainly derived from Ma_3 (Figure S8B). These results indicated that SPP1-CD44 and SPP1-integrin interactions were relatively specific and strong between Ma_3 and FAP subclusters.

*SPP1*/OPN was reported to promote renal fibrosis and bone marrow fibrosis by activating fibroblast or mesenchymal stromal cells.^66,67^ To justify the role of *SPP1*/OPN in fibrotic remodeling of PVAT, we next examined the expression level and spatial distribution in coronary PVAT, respectively. The mRNA level of *SPP1* in coronary PVAT was significantly increased in atherosclerosis compared with NC in both single-cell and bulk level (Figure 6G and 6H). Importantly, the mRNA level of *SPP1* in coronary PVAT was positively related to the fibrosis degree of coronary PVAT, as well as coronary stenosis grade (Figure 6I and 6J). We further explored the spatial distribution of OPN and fibrosis and found that OPN was enriched in the fibrotic area (collagen I positive) in coronary PVAT (Figure 6K; Figure S9). The above results suggested that OPN played an important role in the fibrotic remodeling of PVAT, and targeting OPN-CD44 or OPN-integrin might be a potential strategy to alleviate the fibrotic remodeling of PVAT.

**Figure 7.**
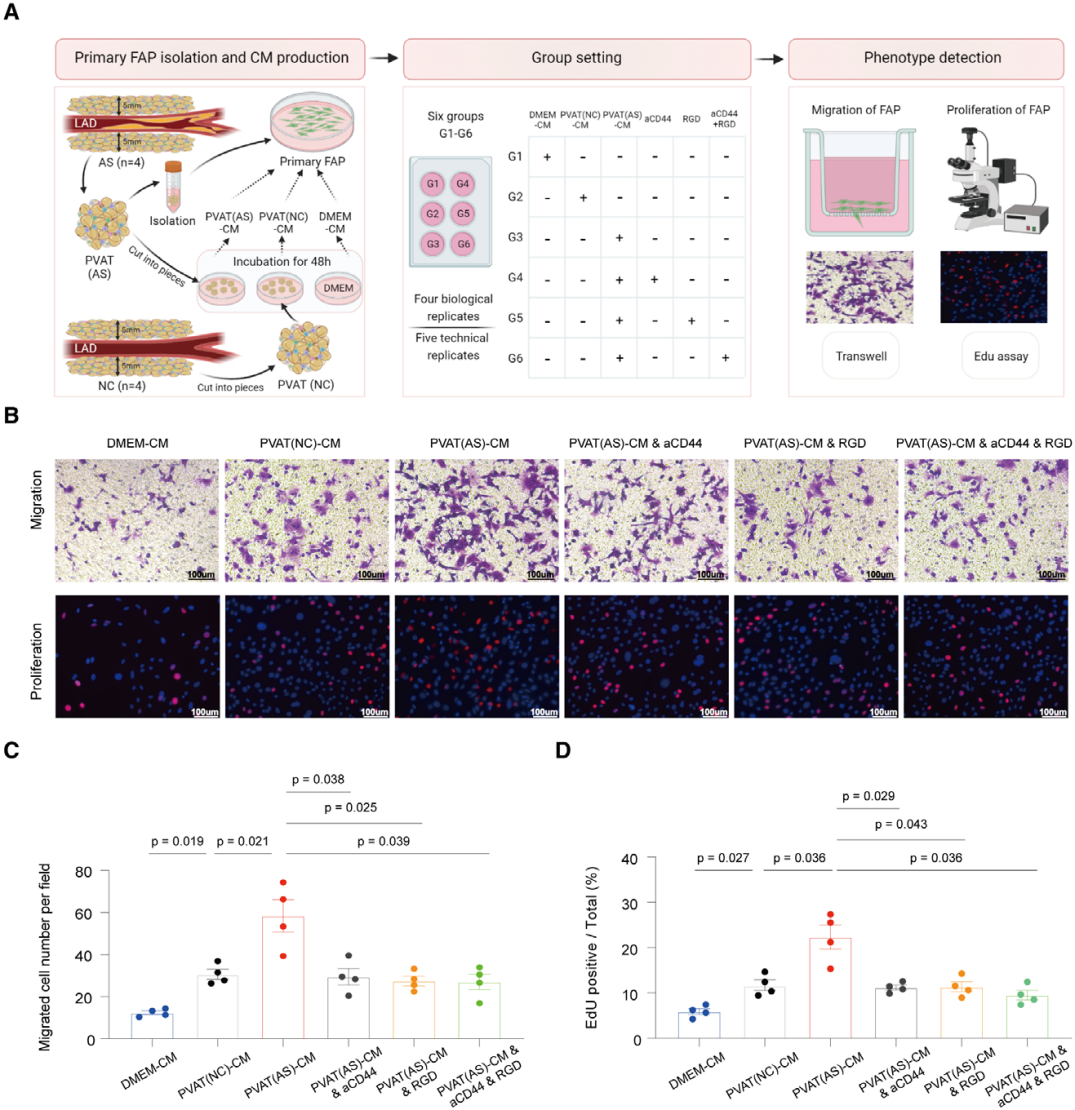
**Conditioned medium from perivascular adipose tissue (PVAT) of patients with atherosclerosis (AS) significantly promotes the fibrogenesis of human fibroadipogenic progenitors (FAPs) through CD44/integrin axis. A**, Schematic representation of the experimental procedure. **B**, Representative images of transwell migration assay (**top**) and EdU (5-ethynyl-2’-deoxyuridine) proliferation assay (**bottom**) of human FAP cells. Scale bar, 100 µm. **C** and **D**, Quantitative analysis of transwell migration assay (**C**) and EdU proliferation assay (**D**) of human FAP cells. Four points in each group in **C** and **D** represent 4 biological replicates. Comparisons of the cell migration or proliferation between each 2 groups were performed using the Student *t* test; *P* values were adjusted for multiple hypothesis testing using the Benjamini-Hochberg method.

Next, we conducted in vitro studies to explore whether targeting OPN-CD44/integrin could attenuate the profibrotic activation of primary FAP cells isolated from human PVAT by detecting 2 important fibrotic phenotypes at the cellular level, namely cellular migration and proliferation (Figure 7A). Compared with NC, CM from PVAT of 4 atherosclerosis patients induced a 1.89-fold and a 1.92-fold increase in FAP migration and proliferation, respectively. But these 2 effects were ameliorated by pretreatment with neutralizing CD44 antibody, Arg-Gly-Asp peptide (RGD peptide, an integrin inhibitor), or cocktail thereof (*P*<0.05; Figure 7B through 7E). Thus, CM from PVAT of atherosclerosis patient contributes to the migration and proliferation of FAP via OPN-CD44/integrin, and OPN is associated with PVAT fibrosis and LAD stenosis degree. Together, our results suggest that inhibition of OPN could serve as a potential target for alleviating the fibrosis of PVAT in atherosclerosis.

## DISCUSSION

**Figure 8.**
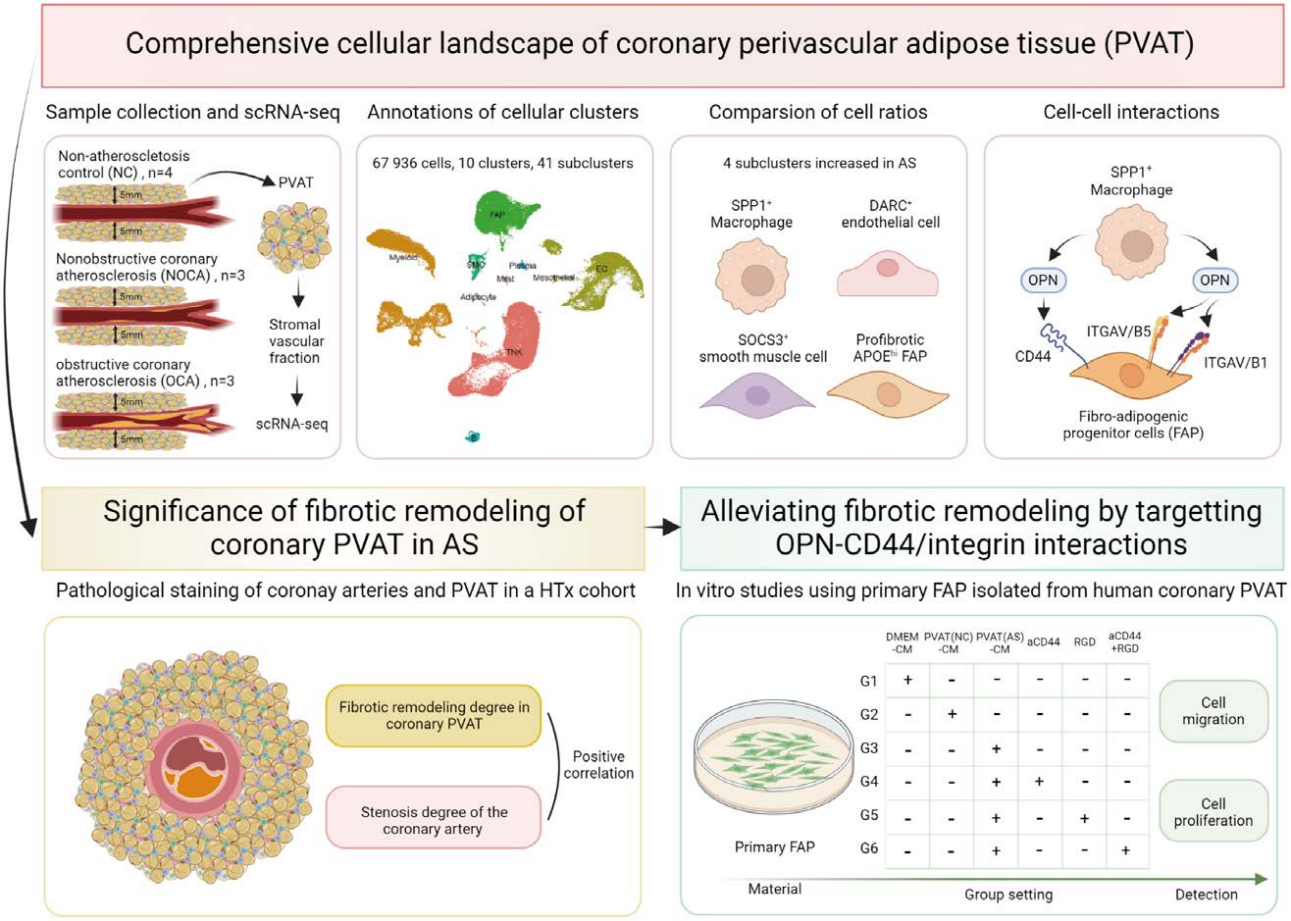
**Summary of the present study.** We constructed a comprehensive cellular landscape of human coronary perivascular adipose tissue from 4 nonatherosclerosis controls, 3 nonobstructive coronary atherosclerosis patients, and 3 obstructive coronary atherosclerosis patients by using single-cell RNA sequencing. We identified the increased cell ratios of *SPP1*^+^ (secreted phosphoprotein 1) macrophage, activated endothelial cell, metabolically activated smooth muscle cell, and profibrosis fibroadipogenic progenitor in the atherosclerosis condition. The fibrosis of coronary perivascular adipose tissue (PVAT) was positively correlated with left anterior descending artery stenosis grade. A potential interaction between *SPP1*^+^ macrophage and fibroadipogenic progenitor was further validated by in vitro study. AS indicates atherosclerosis.

The maintenance of healthy PVAT function is vital for the homeostasis of vessels, and PVAT dysfunction might play a role in the pathogenesis of vascular diseases.^68,69^ Recent studies showed the coronary imaging–derived signatures of PVAT dysfunction could distinguish different stages of CHD and predict the prognosis of patients with CHD, suggesting the association between PVAT dysfunction and atherosclerosis.^6–8^ However, the role of coronary PVAT in pathogenesis of atherosclerosis remains elusive, partly due to 2 reasons. First, it is difficult to obtain human coronary PVAT samples, especially the PVAT surrounding coronary arteries without atherosclerosis or with NOCA. Second, previous studies mainly focused on adipocytes in PVAT, neglecting the importance of SVF cells, which are also related to coronary atherosclerosis.^70,71^ To overcome the above 2 shortcomings, we used scRNA-seq to characterize the transcriptome of the SVF of coronary PVAT from patients at different stages of atherosclerosis (NC, NOCA, and OCA) at the single-cell resolution (Figure 8). To our knowledge, this is the first study for scRNA-seq of coronary PVAT, and data will serve as a powerful resource for future hypothesis-driven investigations into the mechanisms of coronary PVAT plasticity.

Coronary artery and surrounding PVAT mainly interact in the local position^72,73^; therefore, we explored the interaction between a certain vessel and its surrounding PVAT, and the atherosclerotic stages in the study were defined in the level of vessel instead of patient. We choose LAD for our study because it is the most common site for atherosclerosis in the coronary arteries. Thus, the stenosis extent of other epicardial coronary arteries was not provided.

Our characterization of adipose-resident immune cells, representing 59.5% of the cells, confirmed the complex nature of CHD-related coronary PVAT inflammation. We identified 25 different immune cell types including T cells, NK cells, monocytes, macrophages, DCs, B cells, plasma cells, and mast cells.

T cells are critical drivers and modifiers of the pathogenesis of atherosclerosis. CD4^+^ T cells are commonly found in atherosclerotic plaques,^74^ while it is unclear how they act in human coronary PVAT in the atherosclerotic condition. Here, we reported several new findings (Figure 2). First, we found CD4^+^ T cells exhibited anti-inflammatory function in NC condition.^32,33^ Second, in OCA condition, we identified a subpopulation of CD4^+^ T cells expressing metallothionein and HSP, which play a role in adipose tissue dysfunction,^54^ thus opening up a new ground for the possible identification of T-cell roles in adipose inflammation. Techniques such as advanced mRNA sequencing and clinical interventional study are needed to further explore these interesting findings.

Macrophage infiltration into adipose tissue is the key pathological factor leading to adipose tissue dysfunction and atherosclerosis. Resident adipose tissue macrophages exhibit an anti-inflammatory phenotype and promote the development of well-metabolized beige adipose tissue. The increase of proinflammatory macrophages in adipose tissue has a negative impact on adipose tissue function, including inhibiting adipogenesis and promoting inflammation, insulin resistance, and fibrosis.^75^ In our study, some of these roles are found to change during the development of coronary atherosclerosis (Figure 3). We observed that due to the high expression of genes involved in lipid metabolism, although in minority, our macrophage subtype (SPP1^+^ macrophage) may be associated with atherosclerosis. We demonstrated that they exhibited selective expression of CD9,^76^ indicating that they represented a different type of adipose tissue macrophage. For example, Hill et al^77^ reported that mouse CD9 adipose tissue macrophages are lipid loaded and proinflammatory. Further, pseudotime analysis showed that classical monocyte differentiated into resident MHC^hi^ macrophage and the latter differentiate into the SPP1^+^ macrophage. These results provided new insights into adipose tissue macrophage.

The ECs accounted for 19.2% of all cells in coronary PVAT, with distinctive signatures dividing the group into subpopulations. About 25% of ECs were activated (EC_C1; Figure 4), especially in atherosclerosis condition. These activated cells were active in the production of cytokines, indicating the proinflammatory role in coronary atherosclerosis. This is consistent with a study that indicated PVAT inflammation precedes endothelial dysfunction and the formation of atherosclerotic plaque.^78^ Our largest population of ECs (EC_C0) were characterized as microvascular ECs that handle fatty acid. These results are consistent with recent studies that indicate the importance of endothelial fatty acid uptake in adipose tissue, especially during inflammation.^54,79^ Another subtype (EC_C3) was identified as lymphatic vessel ECs. This is interesting because recent studies have shown the importance of cross talk between lymphatic vessel and adipose tissue and that lymphatic dysfunction is associated with metabolic diseases.^80^ We extended the efforts of these studies and found that coronary PVAT contained lymphatic vessel ECs, accounting for almost 10% of ECs.

The FAP cluster contained 7 different subpopulations. FAP_C0 highly expressed APOE and mainly enriched in ECM organization, which indicated a profibrotic role of FAP_C0. Interestingly, in response to atherosclerosis, we observed a significant increase in FAP_C0, indicating that atherosclerosis led to an increase in adipose tissue fibrosis. Fibrotic remodeling is one of the key players in adipose tissue dysfunction and a target to prevent vascular diseases.^81^ Previous studies showed there was less fibrotic remodeling in PVAT surrounding atherosclerosis-resistant vessels (such as the internal thoracic artery and saphenous vein) compared with PVAT surrounding atherosclerosis-susceptible vessels (such as the coronary artery and aorta).^82,83^ However, it was not clear whether the degree of fibrotic remodeling of PVAT surrounding coronary artery changed as atherosclerosis progresses. In this study, we assured the fibrotic remodeling degree was increased in PVAT surrounding atherosclerotic coronary arteries compared with PVAT surrounding nonatherosclerotic coronary arteries and was positively correlated to the degree of coronary stenosis (Figure 5). These results support the role of coronary PVAT fibrosis in coronary atherosclerosis.

We found the increased fibrotic remodeling of PVAT surrounding atherosclerotic coronary arteries was associated with 2 important cellular populations: the fibrosis-stimulating *SPP1*^+^ macrophages and fibrosis-producing FAP cells, both of the 2 cellular populations were increased in PVAT surrounding atherosclerotic coronary arteries. OPN, encoded by *SPP1*, has been reported to promote fibroblast migration, proliferation, and promote fibrosis of several organs.^66,84^ It activates a fibrotic transcriptional program in adipocyte progenitors in obese mouse as well.^85^ Through cell-cell interaction analysis, we inferred that *SPP1*^+^ macrophages and FAP cells communicate via OPN-CD44/integrin (Figure 6). In vitro experiments showed CM from PVAT of patients with CHD induced FAP migration and proliferation, which were 2 important phenotypes of fibrotic remodeling. Therefore, our study reports the potential role of OPN in the coronary PVAT fibrosis. Importantly, blocking the cellular interactions by neutralizing the CD44 antibody or integrin inhibitor RGD peptide could effectively prevent the migration and proliferation of FAP (Figure 7), indicating OPN-CD44/integrin might be a potential target for alleviating fibrosis of PVAT surrounding coronary arteries.

There are 4 main limitations in this study. First, the scRNA-seq data were derived from 4 NC samples, 3 NOCA samples, and 3 OCA samples. The small sample size may limit the statistical power. But we used a validation cohort to further validate the key findings from scRNA-seq data. Second, the patients enrolled in the present study were end-stage heart failure patients, meaning that the cellular landscape in coronary PVAT might be affected by heart failure. However, it does not affect the reliability of the conclusions in the study. Third, control and NOCA patients both were diagnosed with dilated cardiomyopathy, but the OCA patients were diagnosed with ischemic cardiomyopathy. The analysis between OCA and other groups may be affected by the main diagnostic difference of a history of myocardial infarction. Fourth, we have verified the association between fibrotic remodeling of coronary PVAT and atherosclerosis, but the direct causal relationship between them has not been validated, which should be further studied in the future.

## CONCLUSIONS

*SPP1*^+^ macrophages accumulated in coronary PVAT surrounding atherosclerotic coronary arteries, and they promoted the migration and proliferation of FAP cells via the OPN-CD44/integrin interaction.

## ARTICLE INFORMATION

### Acknowledgments

M. Fu and S. Shu conducted the study and wrote the manuscript. W. Feng and J. Song designed and supervised the project. X. Chen supervised the study. H. Cui, Z. Zeng, and Y. Yang performed the data analyses and data integration. Z. Peng assisted in data interpretation and manuscript editing. R. Zhao provided clinical samples and information. X. Wang and X. Liu performed the immunostaining experiments. L. Du and M. Wu assisted in the clustering and annotation of T cells. The authors thank the Chinese Academy of Medical Sciences Innovation Fund for Medical Sciences (2021-I2M-1-064) and the National Natural Science Foundation of China (82125004). The authors also thank the staff and the graduate students of Fuwai Hospital who gave us valuable advice for this project and the support of single-cell RNA sequencing analysis from Intelliphecy, Inc, Shenzhen, China.

### Sources of Funding

This work was funded by grants from the Chinese Academy of Medical Sciences Innovation Fund for Medical Sciences (2021-I2M-1-064), the National Natural Science Foundation of China (82125004, 82000225), the Fundamental Research Funds for the Central Universities (3332022125), the Central Public-Interest Scientific Institution Basal Research Fund of the Chinese Academy of Medical Sciences (2020-PT310-001), Science and Technology Promotion Program of Air Force Medical Center, People’s Liberation Army (2022ZTYB34), and the R&D Program of Beijing Municipal Education Commission (KM202210025005).

### Disclosures

None.

### Supplemental Material

Supplemental Methods

Tables S1–S6

Figures S1–S9

References 53,86–95

Major Resources Table

## Supplementary Material


