# Supplementary material for: Single-Cell RNA Sequencing of Coronary Perivascular Adipose Tissue From End-Stage Heart Failure Patients Identifies SPP1+ Macrophage Subpopulation as a Target for Alleviating Fibrosis
Source: Arterioscler Thromb Vasc Biol. 2023 Sep 14;43(11):2143–64. doi: 10.1161/ATVBAHA.123.319828 (PMC10597444; doi:10.1161/ATVBAHA.123.319828)
Supplement: Supplementary file 1 [file atv-43-2143-s001.pdf]

SUPPLEMENTAL MATERIALS

Single-cell RNA sequencing of coronary perivascular adipose tissue from end-stage heart failure patients identifies *SPP1*+ macrophage subpopulation as a target for alleviating fibrosis

Running title: scRNA-seq; coronary perivascular adipose tissue

Mengxia Fu (付梦霞)[#,1,2], Songren Shu (舒松仁)[#,1,3], Zhiming Peng (彭志明)[4], Xiaorui Liu (刘晓蕊)[1,3], Xiao Chen (陈晓)[1,3], Zhiwei Zeng (曾志威)[1], Yicheng Yang (杨逸成)[1], Hao Cui (崔皓)[1,3], Ruojin Zhao (赵若瑾)[1,3], Xiaohu Wang (王小虎)[1,3], Leilei Du (杜雷雷)[5], Min Wu (吴敏)[2], Wei Feng (凤玮)[*,1,6], Jiangping Song (宋江平)[*,1,3,6,7]

[1]State Key Laboratory of Cardiovascular Disease, Fuwai Hospital, National Center for Cardiovascular Diseases, Chinese Academy of Medical Sciences and Peking Union Medical College, Beijing, China.

[2]Galactophore Department, Galactophore Center, Beijing Shijitan Hospital, Capital Medical University, Beijing, China.

[3]The Cardiomyopathy Research Group at Fuwai Hospital.

[4]Department of Orthopedics, Peking Union Medical College Hospital, Chinese Academy of Medical Sciences and Peking Union Medical College, Beijing, China.

[5]Laboratory of Cardiovascular Science, Beijing Clinical Research Institute, Beijing Friendship Hospital, Capital Medical University, 95 Yongan Road, Beijing, 100050, China.

[6]Department of Cardiovascular Surgery, Fuwai Hospital, National Center for Cardiovascular Diseases, Chinese Academy of Medical Sciences and Peking Union Medical College, Beijing, China.

[7]Shenzhen Key Laboratory of Cardiovascular Disease, Fuwai Hospital Chinese Academy of Medical Sciences, Shenzhen, China.

# These authors contributed equally.

*** Corresponding authors:**

Wei Feng, 167 Beilishi Road, Xicheng District, Beijing, 100037, China.

fengwei@fuwai.com

Jiangping Song, 167 Beilishi Road, Xicheng District, Beijing, 100037, China.

fwsongjiangping@126.com

Methods:

Counts of Different Biotypes

The output of cellranger pipeline resulted in 33,538 genes with ensemble ID. Those genes were annotated with human build 38 (grch38), which contains 40 unique biotypes. Statistics was obtained using R (version 4.05).

Data Integration and Clustering

Canonical correlation analysis (CCA) algorithm was applied to integrate single cell transcriptomic data from different samples.[87] SelectIntegrationFeatures function was used with default parameters to select features to integrate multiple datasets. Integration anchors were identified by FindIntegrationAnchors function. Data integration was performed with IntegrateData function with the identified anchors. The integrated data was scaled using ScaleData function. Dimensionality reduction was performed using PCA, UMAP and TSNE. The top 30 principal components were used in downstream analysis. The nearest neighbors were identified with FindNeighbors function. SingleR (1.10.0) was employed to perform automatic annotation of cellular clusters[88]. The cells were annotated with a reference created with 713 microarray samples from the Human Primary Cell Atlas (HPCA). Each sample has been assigned to one of 37 main cell types ("label.main") . The result of SingleR annotation was compared with our manual annotation. Classical cell type specific markers were used for cross-validation and final assignment of cell identity.

Re-clustering

Subsets of the atlas were re-clustered to obtain finer cellular subtypes, including TNK atlas (T cells and NK cells), Myeloid atlas (Macrophages, Monocytes, DC and Neutraphils), FAP atlas (fibro-adipogenic progenitor) and EMS atlas (EC, MC and SMC). Cells of interest were subset and clustered with FindClusters using different resolution parameter set as 1. The subcluster specific markers were identified with FindAllMarkers function to determine whether resolution should be increased or subclusters should be merged. Finally, TNK atlas is comprised of eight T cell subtypes (T_1, T_2, T_3, T_4, T_5, T_6, T_7, T_8) and three NK cell subtypes (NK_1, NK_2 and NK_3); Myeloid atlas is comprised of five macrophage subtypes (Ma_1, Ma_2, Ma_3, Ma_4, Ma_5), two DC subtype (DC_1, DC_2), and four neutrophil subtypes (N_1, N_2, N_3, N_4); FAP atlas is comprised of seven FAP subtypes (FAP_C0, FAP_C1, FAP_C2, FAP_C3, FAP_C4, FAP_C5, FAP_C6); EMS atlas is comprised of five EC subtypes (EC_C0, EC_C1, EC_C2, EC_C3, EC_C4), one MC subtype and two SMC subtypes(SMC_C0, SMC_C1).

RNA Extraction and qRT-PCR

The protocol for RNA extraction, detection, and reverse transcription has been described previously [89]. Total RNA was isolated from 30 mg liquid-nitrogen-preserved coronary PVAT in using TRIzol reagent according to the manufacturer’s instructions (15596026, Invitrogen). The concentration of isolated RNA was measured using the NanoDrop 2000 (Thermo Fisher Scientific). One microgram RNA was reverse transcribed using the Takara Primescript RT-PCR Kit (RR036A, Takara). Gene expression was detected using SYBR® Select Master Mix (4472908, Applied Biosystems) and the QuantStudio 5 Real-Time PCR System (A25742,

Applied Biosystems). Based on published literatures[90,91], 18S was used as the internal reference for coronary PVAT. Relative gene expression was calculated using the ΔΔCt method. The primer sequences used in this study are listed in **Table S6**.

Table S1. Information of patients enrolled in the study.

	NC_1	NC_2	NC_3	NC_4	NOCA_1	NOCA_2	NOCA_3	OCA_1	OCA_2	OCA_3
**Sex**	Male	Male	Male	Male	Male	Male	Male	Male	Male	Male
**Age, y**	52	46	56	40	62	44	45	48	49	56
**BMI, kg/m^2**	24.8	22.9	23.8	24.6	23.5	22.2	23.5	23.6	22.8	22.2
**Diagnosis**	DCM	DCM	DCM	DCM	DCM	DCM	DCM	ICM	ICM	ICM
**NYHA class**	IV	IV	IV	III	IV	III	IV	III	IV	IV
**Medical history**										
Myocardial infarction	No	No	No	No	No	No	No	Yes	Yes	Yes
Hypertension	No	No	No	No	No	No	No	No	No	No
Diabetes mellitus	No	No	No	No	No	No	No	No	No	No
Smoking status	Past (quit 10y ago)	Past (quit 4y ago)	Never	Never	Past (quit 2y ago)	Never	Current (20y)	Current (15y)	Never	Past (quit 3y ago)
Angina	No	No	No	No	No	No	No	Yes	Yes	Yes
ST-T change on ECG	No	No	No	No	No	No	No	Yes	Yes	Yes
**Laboratory tests**										
hs-CTnI, μg/L (Ref. 0-0.034)	0.032	0.033	0.018	0.025	0.030	0.755	0.045	3.758	1.290	2.201
CTnI, μg/L (Ref. 0-0.08)	0.030	0.017	0.006	0.007	0.020	0.333	0.025	4.060	0.138	0.069
TG, mmol/L (Ref. 0.38-1.76)	1.60	1.61	0.66	0.94	1.11	0.92	1.46	1.72	0.80	0.76
LDL-C, mmol/L (Ref. 0-3.37)	2.860	2.550	2.260	1.330	2.800	2.090	3.770	3.750	1.040	1.240
**Echocardiography**										
LVEF, %	31	30	20	31	30	45	20	27	27	34
LVEDD, mm	72	74	83	75	70	82	80	73	72	75
**LAD calcification in coronary CT**	No	No	No	No	No	Yes	No	Yes	Yes	Yes
**LAD stenosis in CCTA, %**	0	0	0	0	30	30	40	80	90	100
**LAD stenosis in coronary angiography, %**	/	/	/	/	/	/	/	90	100	100

/: The examination has not been performed. NC, non-atherosclerosis control; NOCA, nonobstructive coronary atherosclerosis; OCA, obstructive coronary atherosclerosis; BMI, body mass index; DCM, dilated cardiomyopathy; ICM, ischemic cardiomyopathy; NYHA, New York Heart

Association; ECG, electrocardiography; hs-CTnI, high-sensitivity cardiac troponin I; CTnI, cardiac troponin I; TG, Triglyceride; LDL-C, Low density lipoprotein cholesterol; LVEF, Left ventricular ejection fraction; LAD, left anterior descending branch; LVEDD, left ventricular end-diastolic diameter; CCTA, coronary computed tomography angiography.

Table S2. Summary of clinical characteristics of patients among three groups.

	Single-cell RNA sequencing (n=10)				Validation cohort (n=40)			
	NC (n=4)	NOCA (n=3)	OCA (n=3)	p value	NC (n=16)	NOCA (n=14)	OCA (n=10)	p value
**Male, n (%)**	4 (100.0)	3 (100.0)	3 (100.0)	1.000	12 (75.0)	11 (78.6)	8 (80.0)	1.000
**Age, y**	48.5 ± 3.5	50.3 ± 5.8	51.0 ± 2.5	0.900	49.0 ± 4.7	51.4 ± 9.7	53.8 ±5.2	0.241
**BMI, kg/m^2**	24.0 ± 0.4	23.1 ± 0.4	22.8 ± 0.4	0.181	20.7 ± 4.2	22.9 ± 3.4	21.1± 1.7	0.229
**Diagnosis, n (%)**				**0.017**				**<0001**
DCM	4 (100.0)	3 (100.0)	0 (0.0)		11 (68.8)	9 (64.3)	0 (0.0)	
ICM	0 (0.0)	0 (0.0)	3 (100.0)		0 (0.0)	0 (0.0)	10 (100.0)	
HCM	0 (0.0)	0 (0.0)	0 (0.0)		2 (12.5)	1 (7.1)	0 (0.0)	
RCM	0 (0.0)	0 (0.0)	0 (0.0)		2 (12.5)	1 (7.1)	0 (0.0)	
ARVC	0 (0.0)	0 (0.0)	0 (0.0)		1 (6.3)	1 (7.1)	0 (0.0)	
CHD	0 (0.0)	0 (0.0)	0 (0.0)		0 (0.0)	2 (14.3)	0 (0.0)	
**NYHA class, n (%)**				1.000				0.924
Ⅱ, %	0 (0.0)	0 (0.0)	0 (0.0)		2 (12.5)	1 (7.1)	1 (10.0)	
III, %	1 (25.0)	1 (33.3)	1 (33.3)		4 (25.0)	3 (21.4)	1 (10.0)	
IV, %	3 (75.0)	3 (66.6)	3 (66.6)		10 (62.5)	10 (71.4)	8 (80.0)	
**Medical history**								
Myocardial infarction, n (%)	0 (0.0)	0 (0.0)	3 (100.0)	**0.017**	0 (0.0)	0 (0.0)	10 (100.0)	**<0001**
Hypertension, n (%)	0 (0.0)	0 (0.0)	0 (0.0)	1.000	3 (18.8)	4 (28.6)	3 (30.0)	0.725
Diabetes mellitus, n (%)	0 (0.0)	0 (0.0)	0 (0.0)	1.000	2 (12.5)	2 (14.3)	2 (20.0)	1.000
Angina, n (%)	0 (0.0)	0 (0.0)	3 (100.0)	**0.017**	0 (0.0)	0 (0.0)	10 (100.0)	**<0001**
ST-T change on ECG, n (%)	0 (0.0)	0 (0.0)	3 (100.0)	**0.017**	0 (0.0)	0 (0.0)	10 (100.0)	**<0001**
**Laboratory tests**								
hs-CTnI, μg/L (Ref. 0-0.034)	0.03 (0.02, 0.33)	0.05 (0.03, *)	2.20 (1.30, *)	**0.034**	0.02 (0.01, 0.02)	0.03 (0.02, 0.04)	1.74 (1.24, 2,76)	**<0001**
CTnI, μg/L (Ref. 0-0.08)	0.01 (0.01, 0.03)	0.03 (0.02, *)	0.14 (0.07, *)	0.066	0.04 (0.02, 0.05)	0.05 (0.02, 0.07)	0.70 (0.37, 1.37)	**<0001**
TG, mmol/L (Ref. 0.38-1.76)	1.2 ± 0.2	1.2 ± 0.2	1.1 ± 0.3	0.951	1.1 ± 0.4	1.0 ± 0.4	1.1 ± 0.5	0.836
LDL-C, mmol/L (Ref. 0-3.37)	2.3 ± 0.3	2.8 ± 0.5	2.0 ± 0.9	0.575	2.3 ± 0.4	2.5 ± 0.6	2.4 ± 0.9	0.664
**Echocardiography**								
LVEF, %	28.0 ± 2.7	31.7 ± 7.3	29.3 ± 2.3	0.834	30.1 ± 11.6	28.1 ± 5.9	26.8 ± 4.2	0.488

LVEDD, mm	76.0 ± 4.8	77.3 ± 6.4	73.3 ± 1.5	0.596	64.5 ± 5.6	65.3 ± 4.8	63.3 ± 3.6	0.621
**LAD calcification in coronary CT, n (%)**	0 (0.0)	1 (33.3)	3 (100.0)	**0.033**	0 (0.0)	7 (50.0)	7 (70.0)	**<0001**
**LAD stenosis in CCTA, %**	0 (0, 0)	30 (30, *)	90 (80, *)	**0.014**	0 (0, 0)	30 (15, 35)	82 (75, 96)	**<0001**
**LAD stenosis in coronary angiography, %**	/	/	96.6 ± 3.3	#	/	/	90.0 ± 2,7	#
**Ventricular assist device before HTx, n (%)**	0 (0.0)	0 (0.0)	0 (0.0)	1.000	0 (0.0)	0 (0.0)	0 (0.0)	1.000
**Chronic inotropic therapy before HTx, n (%)**	0 (0.0)	0 (0.0)	0 (0.0)	1.000	0 (0.0)	0 (0.0)	0 (0.0)	1.000

Continuous variables were presented as mean ± standard deviation or median (25th percentile, 75th percentile) based on whether the variables followed normal distribution. Categorical variables were presented as frequency (percentages). Comparisons among the three groups were performed by the analysis of variance (for continuous variables that met the assumptions of normality and homogeneous variance), Kruskal-Wallis test (for continuous variables that did not meet the assumptions of normality), or Fisher's exact test (for categorical variables). SPSS 26.0 (IBM, USA) was used for statistical analysis. NC, non-atherosclerosis control; NOCA, nonobstructive coronary atherosclerosis; OCA, obstructive coronary atherosclerosis; BMI, body mass index; DCM, dilated cardiomyopathy; ICM, ischemic cardiomyopathy; HCM, hypertrophic cardiomyopathy; RCM, restrictive cardiomyopathy; ARVC, arrhythmogenic right ventricular cardiomyopathy; CHD, congenital heart disease; NYHA, New York Heart Association; ECG, electrocardiography; hs-CTnI, high-sensitivity cardiac troponin I; CTnI, cardiac troponin I; TG, Triglyceride; LDL-C, Low density lipoprotein cholesterol; LVEF, Left ventricular ejection fraction; LAD, left anterior descending branch; LVEDD, left ventricular end-diastolic diameter; CCTA, coronary computed tomography angiography HTx, heart transplantation.*, 75th percentile could not be calculated because the limited number of subject in the group of NOCA and OCA (n=3); #, p value could not be calculated because of missing data.

Table S3. Summary of the single-cell RNA sequencing data before and after quality control.

		NC_1	NC_2	NC_3	NC_4	NOCA_1	NOCA_2	NOCA_3	OCA_1	OCA_2	OCA_3
Before quality control	UMI count (Mean)	4,918	6,664	4,518	6,058	5,440	4,014	6,120	6,279	6,489	4,291
	UMI count (Median)	3,405	4,276	3,497	2,720	3,648	2,671	4,137	4,912	3,460	2,861
	Gene count (Mean)	1,612	2,049	1,519	1,720	1,634	1,281	1,946	1,902	1,722	1,440
	Gene count (Median)	1,390	1,739	1,370	1,055	1,314	1,157	1,617	1,672	1,288	1,227
	Mitochondria gene percent (Mean)	5	6	5	15	6	4	7	6	5	6
	Mitochondria gene percent (Median)	3	4	4	10	5	3	5	5	3	5
	Cell count	8192	9228	8245	10933	10596	6415	12924	10313	8242	12626
After quality control	UMI count (Mean)	4,084	5,047	3,976	5,717	4,338	3,094	5,103	5,590	4,541	3,399
	UMI count (Median)	3,275	3,927	3,393	4,186	3,451	2,501	3,880	4,687	2,935	2,708
	Gene count (Mean)	1,489	1,816	1,427	1,930	1,482	1,174	1,777	1,813	1,452	1,307
	Gene count (Median)	1,347	1,630	1,339	1,732	1,255	1,106	1,519	1,619	1,144	1,194
	Mitochondria gene percent (Mean)	3	5	5	8	5	3	6	5	4	6
	Mitochondria gene percent (Median)	3	4	4	8	5	2	5	5	3	5
	Cell count	6,966	6,948	6,902	4,217	7,673	5,541	10,225	6,480	6,265	6,719

NC, non-atherosclerosis control; NOCA, nonobstructive coronary atherosclerosis; OCA, obstructive coronary atherosclerosis; UMI, unique molecular identifier.

Table S4. Counts of different biotypes detected in coronary PVAT of human by scRNA-seq.

Biotype	n
protein_coding	19680
lncRNA	12339
microRNA	386
IG_V_pseudogene	187
IG_V_gene	145
TR_V_gene	106
TR_J_gene	79
transcribed_unprocessed_pseudogene	46
IG_D_gene	37
TR_V_pseudogene	33
transcribed_unitary_pseudogene	19
IG_J_gene	18
IG_C_gene	14
IG_C_pseudogene	9
transcribed_processed_pseudogene	8
TR_C_gene	6
TR_D_gene	4
TR_J_pseudogene	4
IG_J_pseudogene	3
unprocessed_pseudogene	2
processed_pseudogene	1
translated_unprocessed_pseudogene	1
unitary_pseudogene	1
artifact	17
NA	393

Table S5. Cell types assignment by using SingleR and manual annotation.

Clusters	SingleR	Manual	Markers	References
**0**	**Smooth_muscle_cells**	**Fibro-adipogenic progenitor cells**	DCN, LUM, PDGRFA	[92]
1	T_cells	T/NK cells	CD3D, CD52, NKG7, GNLY	[93-95]
2	T_cells	T/NK cells	CD3D, CD52, NKG7, GNLY	[93-95]
3	Neutrophils	Myeloid	LYZ, CD68, S100A8, CXCR2, CD74	[54,55,93,96]
4	Endothelial_cells	Endothelial cells	VWF, PECAM	[55,93,96]
5	Endothelial_cells	Endothelial cells	VWF, PECAM	[55,93,96]
6	Neutrophils	Myeloid	LYZ, CD68, S100A8, CXCR2, CD74	[54,55,93,96]
7	Monocyte	Myeloid	LYZ, CD68, S100A8, CXCR2, CD74	[54,55,93,96]
8	Macrophage	Myeloid	LYZ, CD68, S100A8, CXCR2, CD74	[54,55,93,96]
9	NK_cell	T/NK cells	CD3D, CD52, NKG7, GNLY	[93-95]
10	T_cells	T/NK cells	CD3D, CD52, NKG7, GNLY	[93-95]
11	Endothelial_cells	Endothelial cells	VWF, PECAM	[55,93,96]
12	NK_cell	T/NK cells	CD3D, CD52, NKG7, GNLY	[93-95]
**13**	**Smooth_muscle_cells**	**Fibro-adipogenic progenitor cells**	DCN, LUM, PDGRFA	[92]
14	Smooth_muscle_cells	Smooth muscle cells	ACTA2, MYH11	[93,96]
15	Endothelial_cells	Endothelial cells	VWF, PECAM	[55,93,96]
**16**	**Smooth_muscle_cells**	**Fibro-adipogenic progenitor cells**	DCN, LUM, PDGRFA	[92]
17	Endothelial_cells	Endothelial cells	VWF, PECAM	[55,93,96]
18	B_cell	B cells	CD79A, IGHM	[55]

19	Monocyte	Myeloid	LYZ, CD68, S100A8, CXCR2, CD74	[54,55,93,96]
**20**	**Monocyte**	**Plasma cells**	JCHAIN, IGHA1	[93]
**21**	**Smooth_muscle_cells**	**Adipocyte**	LEP, PLIN	[97]
**22**	**NK_cell**	**Mast cells**	HPGD, KIT	[93]
**23**	**NK_cell**	**T/NK cells**	CD3D, CD52, NKG7, GNLY	[93-95]
**24**	**Smooth_muscle_cells**	**Mesothelial cells**	ITLN1, UPK3B	[92]

After using the SingleR for unbiased clustering, we manually checked the annocations and found that SingleR's definitions for some clusters were incorrect. For these clusters (which are marked in bold), we modified the annotations according to reported literatures.

Table S6. Sequences of primer pairs used for qRT-PCR.

Abbreviation	Gene name	Forward primer (5'-3')	Reverse primer (5'-3')
18S	18S ribosomal RNA	CGCCGCTAGAGGTGAAATTCT	CATTCTTGGCAAATGCTTTCG
SPP1	Secreted phosphoprotein 1	AGGCTGATTCTGGAAGTTCTGAGG	GACTTACTTGGAAGGGTCTGTGGG
Col1A1	Collagen type I alpha 1 chain	CCAGAAGAACTGGTACATCAGCA	CGCCATACTCGAACTGGAAT
Col1A2	Collagen type I alpha 2 chain	GATGTTGAACTTGTTGCTGAGG	TCTTTCCCCATTCATTTGTCTT
Col3A1	Collagen type III alpha 1 chain	CTTCTCTCCAGCCGAGCTTC	TGTGTTTCGTGCAACCATCC
Col4A1	Collagen type IV alpha 1 chain	GCAAATGTGACTGCCATGGA	GAAACCCAATGACACCTTGTAACC
Col4A2	Collagen type IV alpha 2 chain	CCTGAAGGCACAGCTAACCA	TGCTGTTGTCTCGTCTGTCC
Col6A1	Collagen type VI alpha 1 chain	CCCGTGGACCTGTTCTTTGT	CACAGCGGTAGTACCTGTCC
FN1	Fibronectin 1	CTGGCCGAAAATACATTGTAAA	CCACAGTCGGGTCAGGAG
VIM	Vimentin	ACCAACGACAAAGCCCGCCT	CAGATACGCATTGTCAACATCCTGT

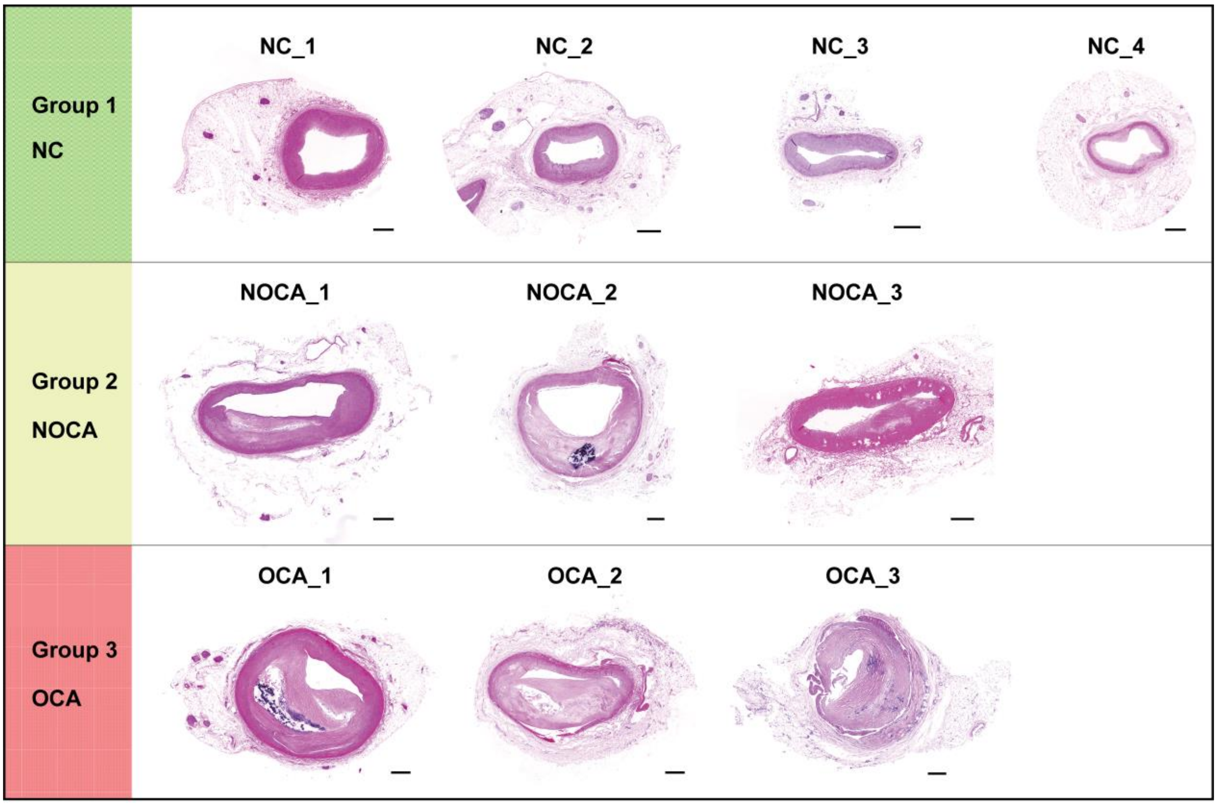

Figure S1. Histological examination of coronary arteries corresponding to each collected coronary PVAT. Scale bar indicates 500μm. NC, non-atherosclerosis control; NOCA, nonobstructive coronary atherosclerosis; OCA, obstructive coronary atherosclerosis.

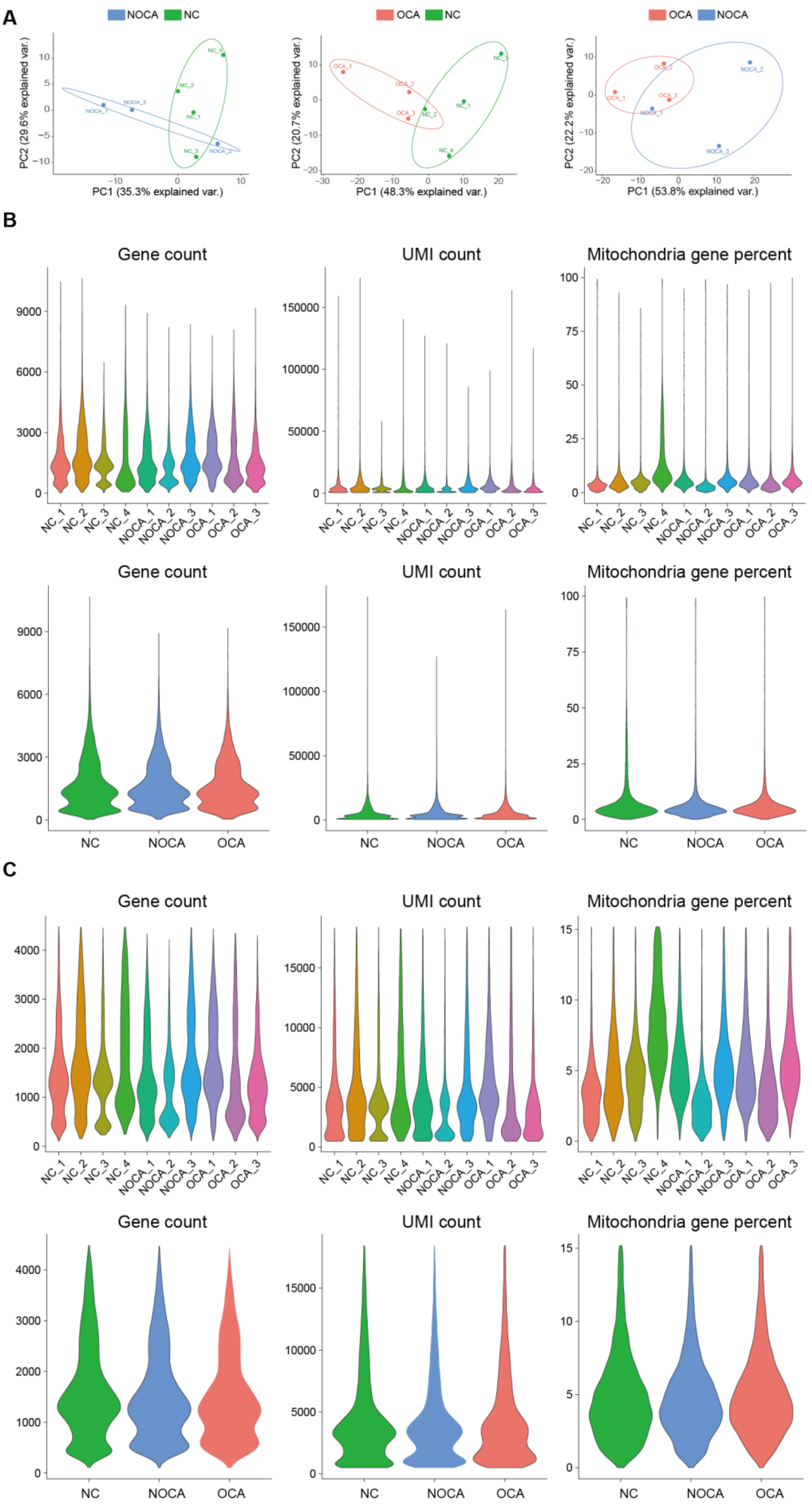

Figure S2. Quality control for scRNA-seq datasets. A. Principal component analysis (PCA)

of the scRNA-seq dataset for PVAT samples. (1) Two-component PCA for scRNA-seq dataset of NOCA and NC (left); (2) OCA and NC (medium); (3) NOCA and OCA (right). Percentages represent variance captured by each principal components 1 and 2 in each analysis. **B-C.** Feature_RNA number, Count_RNA number and mitochondrial RNA percentage of cells in the three groups of PVAT and in 10 samples before (**B**) and after (**C**) quality control. PCA, principal component analysis; NC, non-atherosclerosis control; NOCA, nonobstructive coronary atherosclerosis; OCA, obstructive coronary atherosclerosis.

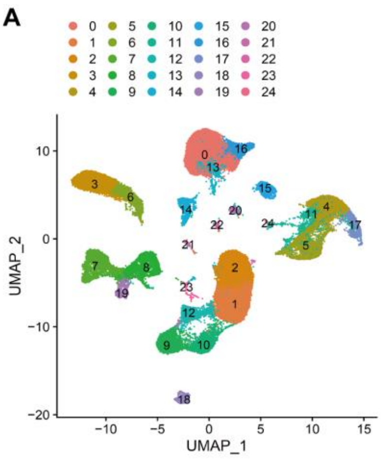

B

Major celltypes	Clusters	Canonical markers
TNK	1,2,9,10,12,23	CCL5, CD52
Myeloid	3,6,7,8,19	S100A8, CD68
EC	4,5,11,15,17	VWF, PECAM1
FAP	0,13,16	PDGFRA, COL1A1
SMC	14	ACTA2, MYH11
B	18	CD79a, IGHD
Plasma	20	JCHAIN
Adipocyte	21	LEP
Mast cell	22	KIT
Mesothelial cell	24	UPK3B

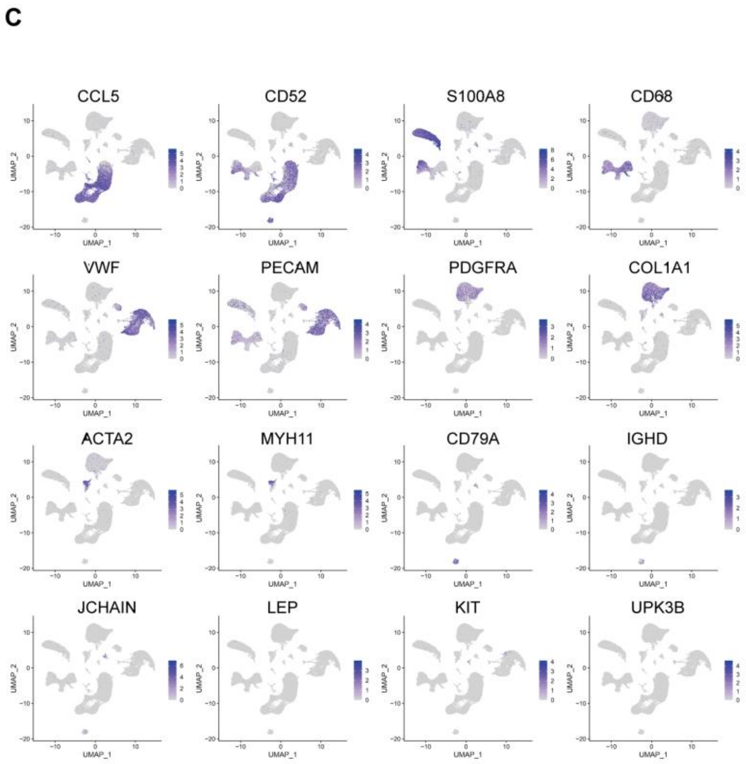

Figure S3. Integrative analysis of 10 samples and initial cluster identification. A, All cells from 10 samples are projected onto a UMAP plot and are colored according to the initially identified 25 clusters (0-24). **B,** A list showing the classical marker genes across the initially identified 25 clusters. **C,** Relative expression of canonical marker genes in all cells projected onto UMAP plots. UMAP, Uniform Manifold Approximation and Projection; NK, natural killer; EC, endothelial cell; SMC, smooth muscle cell; FAP, fibro-adipogenic progenitor.

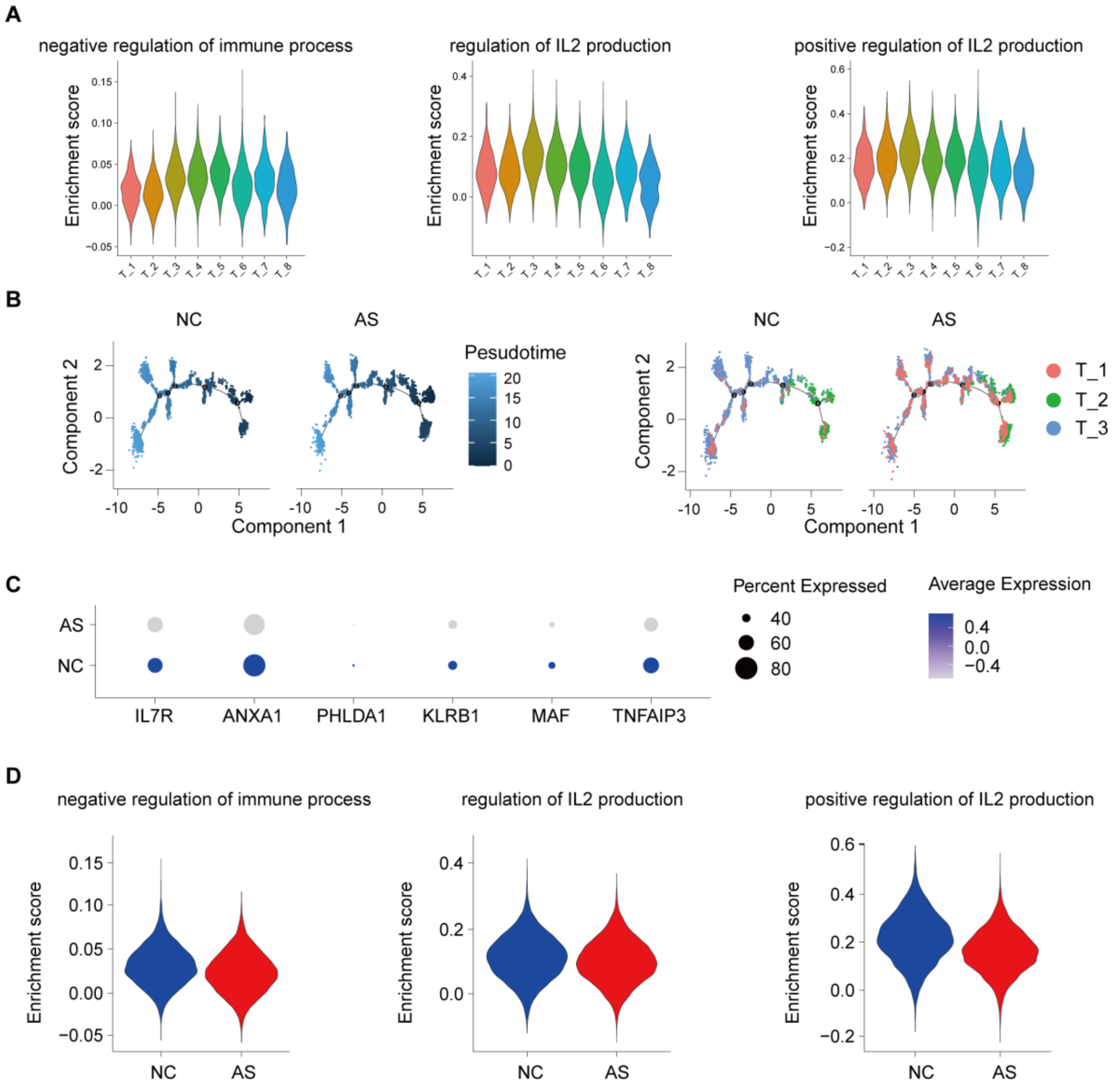

Figure S4. T cells in human perivascular adipose tissue. A, The enrichment scores of anti-inflammatory biological processes in each T subcluster. **B,** Monocle2 analysis of T1, T2 and T3 cells. **C,** Differences in anti-inflammatory genes per CD4$^+$ T cell between NC and AS. **D,** The enrichment scores of anti-inflammatory biological processes in CD4$^+$ T cells between NC and AS. NC, non-atherosclerosis control; AS, atherosclerosis.

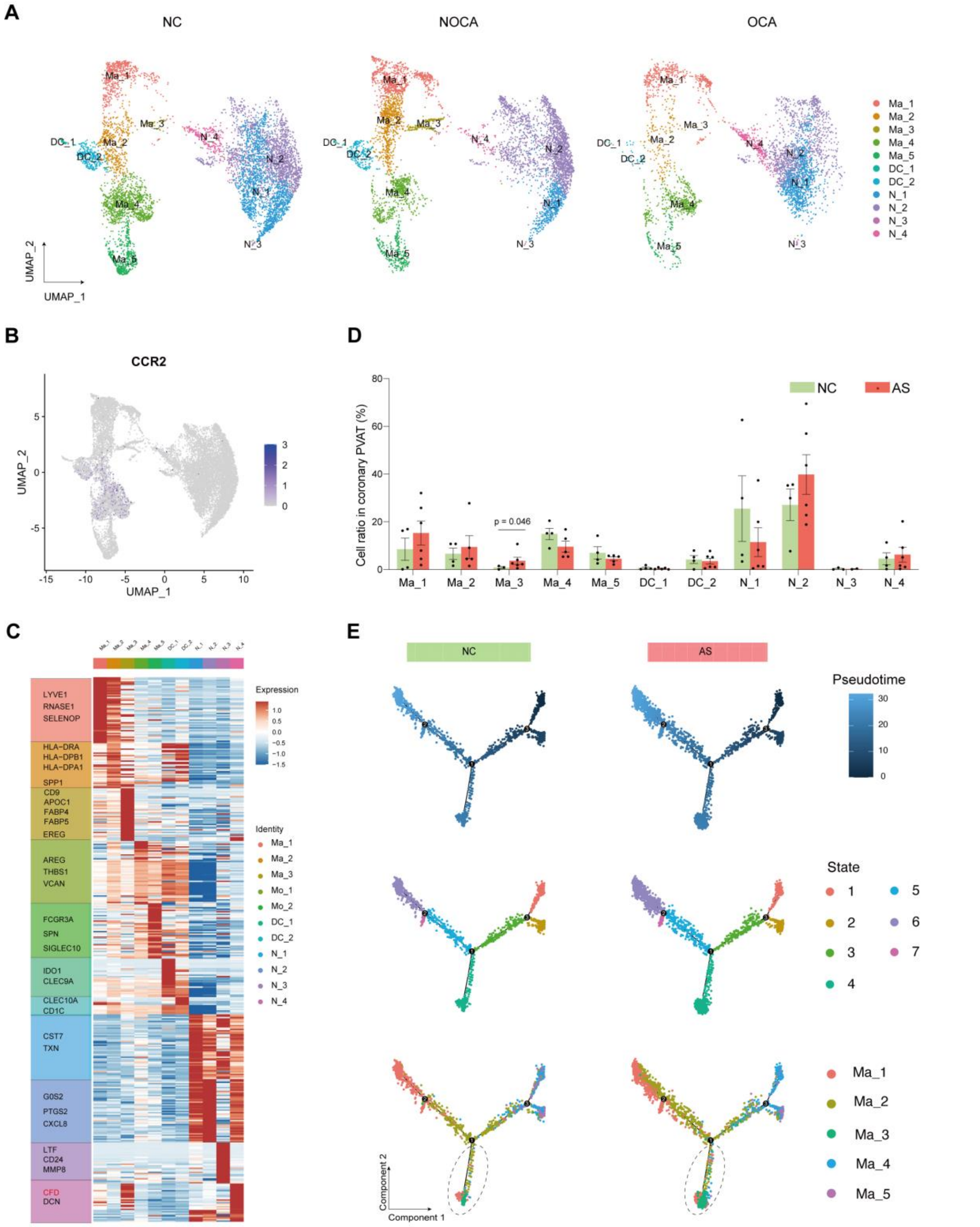

Figure S5. Myeloid clusters in human perivascular adipose tissue. A, Group separated UMAP plot of all myeloid cells colored according to cluster. **B,** UMAP plot of the expression of CCR2 in all myeloid cells. **C,** Heatmap plot of top marker genes in myeloid clusters. **D,** Percentage change tendency of each myeloid cluster during the progression of AS. Student's t-

test with Welch's correction (for Ma_3 cells) was performed to compare the log-transformed proportion of cell subpopulations between each two groups, P values were adjusted for multiple hypothesis testing using the Benjamini-Hochberg method. **E**, Trajectory analysis of macrophage clusters using Monocle2. NC, non-atherosclerosis control; NOCA, nonobstructive coronary atherosclerosis; OCA, obstructive coronary atherosclerosis. UMAP, Uniform Manifold Approximation and Projection.

A

Primary antibody	Isotype control	Concentration	TSA-fluor	Pseudo color
ATCA2	Mouse IgG2a	10 µg/ml	520	Green
DARC	Rabbit IgG	6 µg/ml	520	Green

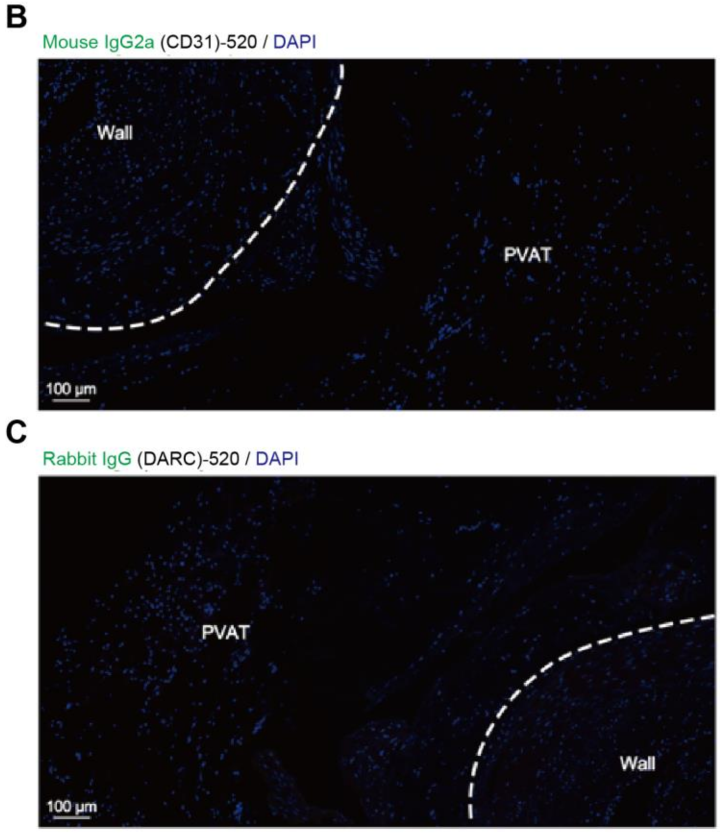

Figure S6. Negative control results for Opal multicolor IHC staining of ACTA2 and DARC. A, Summary of isotype- and concentration-matched isotype controls used for seven primary antibodies used in Opal multicolor IHC staining. **B,** Pictures of negative control results for Opal multicolor IHC staining of ACTA2. **C,** Pictures of negative control results for Opal multicolor IHC staining of DARC. TSA, Tyramide Signal Amplification.

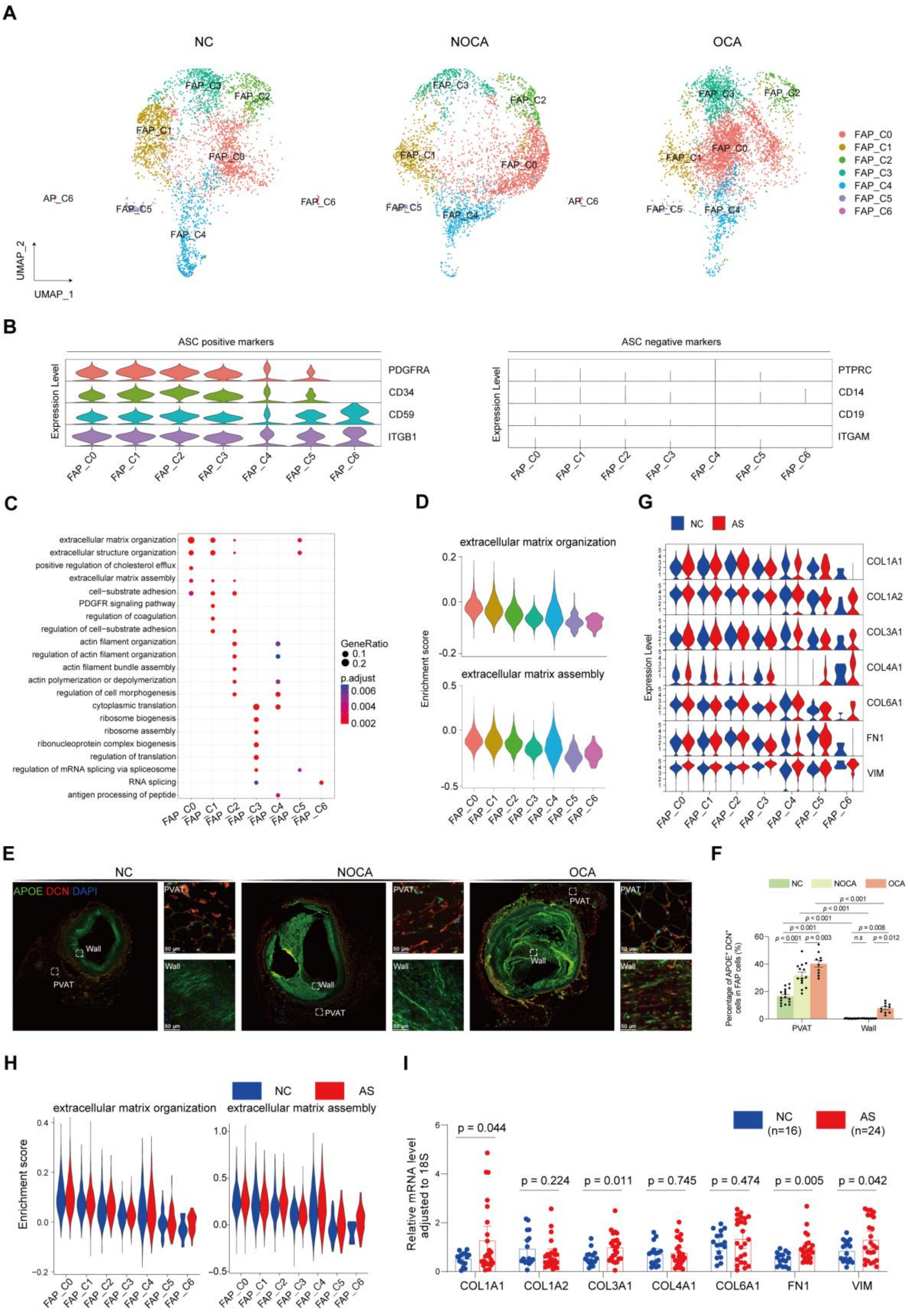

Figure S7. FAP clusters in human perivascular adipose tissue. A, Group separated UMAP plot of all FAP cells colored according to cluster. **B,** Violin plot of ASC positive and negative

markers among each FAP cluster. **C,** The top five enriched GOBP of each FAP cluster. **D,** Enrichment score of ECM organization and assembly among each FAP cluster. **E,** Multiple labeling staining for *APOE*$^{+}$ *DCN*$^{+}$ FAP cells; scale bar indicates 100μm**. F,** Quantification of (E) cell ratio per image (n = 16 in NC, 14 in NOCA, and 10 in OCA). The Mann–Whitney U test was performed to compare the log-transformed proportion of cell subpopulations between each two groups. **G,** Violin plot of fibrotic genes in FAP clusters between NC and AS. **H,** Enrichment score of ECM organization and assembly among each FAP cluster between NC and AS. **I,** Relative mRNA level of several fibrosis genes in the coronary PVAT from NC or AS. NC, non-atherosclerosis control; AS, atherosclerosis; UMAP, Uniform Manifold Approximation and Projection; ECM, extracellular matrix.

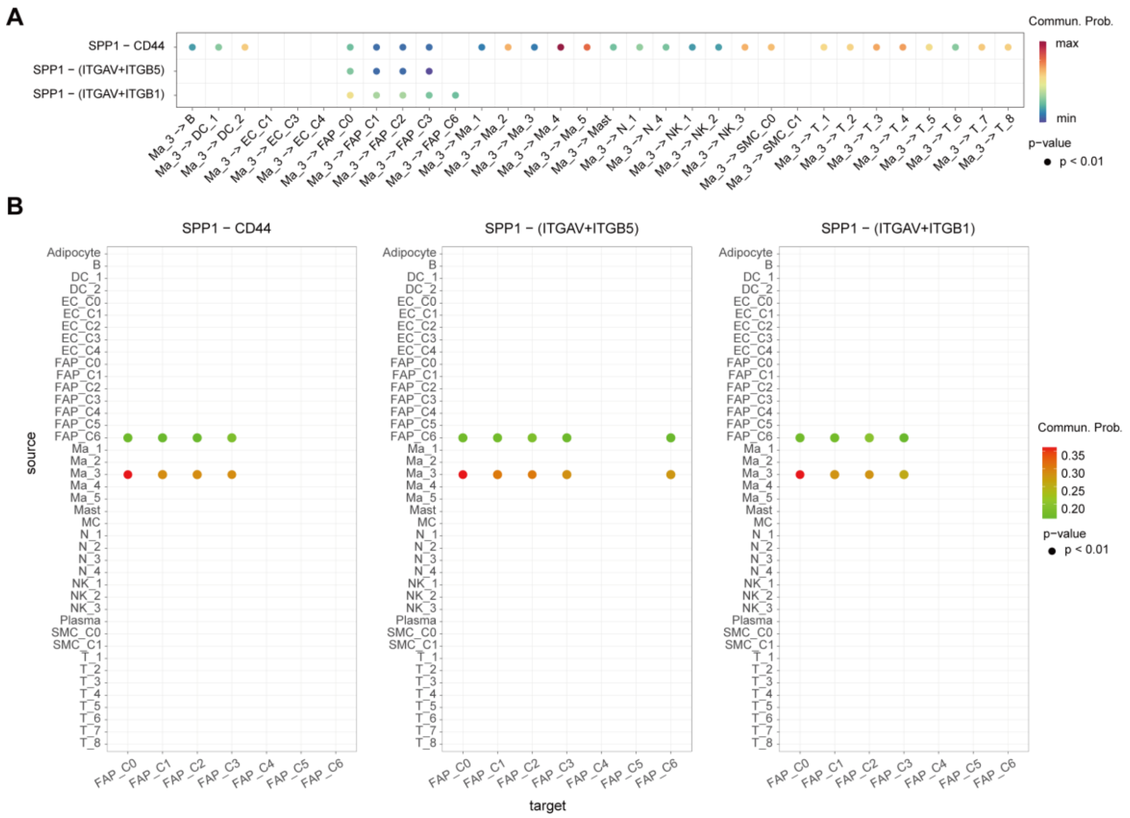

Figure S8. The interaction of OPN signaling between Ma_3 and all cell clusters **(A)**, and between all cell clusters and FAP clusters **(B).**

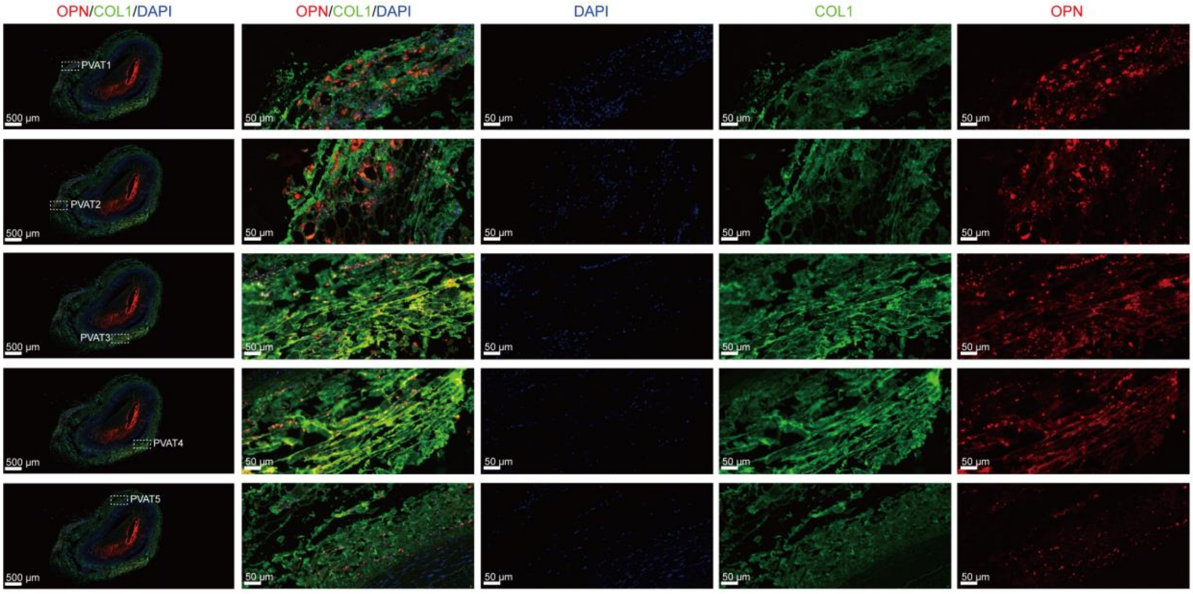

Figure S9. Five different area of OPN (red) and COL (green) immunostaining picture of human coronary PVAT. OPN, osteopontin; COL, collagen.

Major Resources Table

In order to allow validation and replication of experiments, all essential research materials listed in the Methods should be included in the Major Resources Table below. Authors are encouraged to use public repositories for protocols, data, code, and other materials and provide persistent identifiers and/or links to repositories when available. Authors may add or delete rows as needed.

Animals (in vivo studies)

Species	Vendor or Source	Background Strain	Sex	Persistent ID / URL
None				

Genetically Modified Animals

	Species	Vendor or Source	Background Strain	Other Information	Persistent ID / URL
**Parent - Male**	None				
**Parent - Female**	None				

Antibodies

Target antigen	Vendor or Source	Catalog #	Working concentration	Lot # (preferred but not required)	Persistent ID / URL
SPP1	Abcam	ab218237	4 µg/ml		https://www.abcam.com/products/primary-antibodies/osteopontin-antibody-epr21138-ab218237.html
ACTA2	Abcam	ab7817	10 µg/ml		https://www.abcam.com/alpha-smooth-muscle-actin-antibody-1a4-ab7817.html
CD68	ZSGB-BIO	ZM-0060	10 µg/ml		http://www.zsbio.com/product/ZM-0060
CD31	ZSGB-BIO	ZA-0568	6 µg/ml		http://www.zsbio.com/product/ZA-0568
SOCS3	Abcam	ab280884	6 µg/ml		https://www.abcam.com/products/primary-antibodies/socs3-antibody-epr24090-74-ab280884.html
COL1	Abcam	ab138492	8 µg/ml		https://www.abcam.com/products/primary-antibodies/collagen-i-antibody-epr7785-ab138492.html
DARC	Abcam	ab137044	6 µg/ml		https://www.abcam.com/products/primary-antibodies/darc-antibody-epr5205-ab137044.html
APOE	Proteintec	18254-1-AP	6 µg/ml		https://www.ptglab.com/products/APOE-Antibody-18254-1-AP.htm
DCN	Abcam	ab268048	2 µg/ml		https://www.abcam.com/products/primary-antibodies/decorin-antibody-dcn3523-ab268048.html

Mouse IgG2a	abcam	ab18415	10 μg/ml		https://www.abcam.cn/mouse-igg2a-kappa-monoclonal-mg2a-53-isotype-control-ab18415.html
Rabbit IgG	abcam	ab172730	6 μg/ml		https://www.abcam.cn/rabbit-igg-monoclonal-epr25a-isotype-control-ab172730.html

DNA/cDNA Clones

Clone Name	Sequence	Source / Repository	Persistent ID / URL
None			

Cultured Cells

Name	Vendor or Source	Sex (F, M, or unknown)	Persistent ID / URL
Fibro-adipogenic progenitor cells	Human coronary adipose tissue	Male	/

Data & Code Availability

Description	Source / Repository	Persistent ID / URL
Processed gene expression data of 10 perivascular adipose tissue	Gene Expression Omnibus	GSE233870
Sequencing data of 10 perivascular adipose tissue	Genome Sequence Archive	HRA004696
R code notebooks for single-cell RNA-seq data analysis	Non-public data, available upon request	

Other

Description	Source / Repository	Persistent ID / URL
None		

ARRIVE GUIDELINES

The ARRIVE guidelines (https://arriveguidelines.org/) are a checklist of recommendations to improve the reporting of research involving animals. Key elements of the study design should be included below to better enable readers to scrutinize the research adequately, evaluate its methodological rigor, and reproduce the methods or findings.

Study Design

Groups	Sex	Age	Number (prior to experiment)	Number (after termination)	Littermates (Yes/No)	Other description
Group 1	None					

(Control)						
Group 2	None					
Add more if needed						

Sample Size: Please explain how the sample size was decided Please provide details of any a *prior* sample size calculation, if done.

None

Inclusion Criteria

None

Exclusion Criteria

None

Randomization

None

Blinding

None
